# Give spontaneity and self-discovery a chance in ASD: spontaneous peripheral limb variability as a proxy to evoke centrally driven intentional acts

**DOI:** 10.3389/fnint.2013.00046

**Published:** 2013-07-24

**Authors:** Elizabeth B. Torres, Polina Yanovich, Dimitris N. Metaxas

**Affiliations:** ^1^Rutgers University Psychology Department, Computational Biomedicine Imaging and Medicine Center, Rutgers University Center for Cognitive ScienceNew Brunswick, NJ, USA; ^2^Rutgers University Computer Science Department, Rutgers University Center for Cognitive Science PiscatawayNJ, USA; ^3^Rutgers University Computer Science Department, Computational Biomedicine Imaging and Medicine Center, Rutgers University Center for Cognitive SciencePiscataway, NJ, USA

**Keywords:** autism spectrum disorders, proprioceptive feedback, kinesthetic perception, stochastic processes, stochasticity, predictive coding, reliability, motor learning and control, child development, interface

## Abstract

Autism can be conceived as an adaptive biological response to an early unexpected developmental change. Under such conceptualization one could think of emerging biological compensatory mechanisms with unique manifestations in each individual. Within a large group of affected people this would result in a highly heterogeneous spectral disorder where it would be difficult to tap into the hidden potentials of any given individual. A pressing question is how to treat the disorder while harnessing the capabilities and predispositions that the individual has already developed. It would indeed be ideal to use such strengths to accelerate the learning of self-sufficiency and independence, important as the person transitions into adulthood. In this report, we introduce a new concept for therapeutic interventions and basic research in autism. We use visuo-spatial and auditory stimuli to help augment the physical reality of the child and sensory-substitute corrupted kinesthetic information quantified in his/her movement patterns to help the person develop volitional control over the hand motions. We develop a co-adaptive child-computer interface that closes the sensory-motor feedback loops by alerting the child of a cause-effect relationship between the statistics of his/her real-time hand movement patterns and those of external media states. By co-adapting the statistics of the media states and those of the child's real-time hand movements, we found that without any food/token reward the children naturally remained engaged in the task. Even in the absence of practice, the learning gains were retained, transferred and improved 2–4 weeks later. This new concept demonstrates that individuals with autism do have spontaneous sensory-motor adaptive capabilities. When led to their self-discovery, these patterns of spontaneous behavioral variability (SBV) morph into more predictive and reliable intentional actions. These can unlock and enhance exploratory behavior and autonomy in the individual with autism spectrum disorders (ASD).

Each destiny-errored, differently-wired but “cares-to-learn”

child needs assurance they are not weeds

but fragrant flowers to be greeted as valuable.

There in either the tested room or the classroom,

best tell each cherub that they can lead.

Say that they are the guiders to test the best ways to heat their

versed, vested, vastly valuable, vellum varied, esteemed equated

equal, red news never viewed, volumed voices.

When children know their differences will be supported by you

saying you will never stop trying ways to help them find their very

best voice, their fears rest.

There, they are not awed by pity. There, esteem is greeted.

I'm in peace because someone saw all people are real and deserve

being supported to communicate their truths.

Peyton Goddard (Goddard et al., 2012), 65, 69

## Introduction

*How can a learner who does not know what there is to learn manage to learn anyway?* (Thelen, 1994; Smith, 2006). As early as 3 months of age, before reaching or pointing fully matures, typically developing (TD) infants can learn to coordinate their legs and using their own physical movements self-discover coordination patterns that lead them to systematically attain a goal—a goal that has not been instructed or commanded (Thelen and Fisher, 1983a,b; Rovee-Collier, 1989).

Infants seem to have an inherent ability to self-discover goal-directness (Von Hofsten, 1982; Thelen et al., 1993, 1996; van der Meer et al., 1995; Von Hofsten, 2004; Heathcock et al., 2005; Bhat and Galloway, 2006, 2007; Bhat et al., 2007; Lee et al., 2008; van Wermeskerken et al., 2011). It is not known what underlies these abilities and whether they might also exist at a later stage of life in children with developmental delays or in those with behavioral manifestations that lead to a diagnosis of autism spectrum disorders (ASD). The very fact of surviving an early developmental glitch and being able to function in the world despite many developmental challenges, strongly suggests that these children are capable of creating -on their own- compensatory mechanisms that bypass corrupted sensory signals. Could we use adaptive capabilities already present in children and adolescents with a diagnosis of ASD to evoke the self-regulation of goal-directness and intentionality in their actions?

Not all physical movement segments in our actions are goal-directed or performed with the same level of intent (Torres, 2011). A large portion of our acts are spontaneous in nature, occurring beneath our full awareness. These action segments have spontaneous behavioral variability (SBV). This type of variability examined in isolation seems random and noisy, a kind of “nightmare” for researchers, who often try to get rid of it and conform to parametric models assuming a theoretical normal distribution, often without actually examining the statistical distributions inherently present in the experimental data. In order to study the structures inherent to SBV we have designed a new statistical platform for personalized behavioral analyses (SPBA) (Torres and Jose, 2012), which we use in this report to characterize limb motor variability from the periphery in a radically different way from current traditional methods (to be precisely explained in the Methods and Apparatus section of this report).

The SBV has not been widely explored in ASD motor research. The focus has rather been on goal-directed behaviors where the targets are explicitly defined, or where the child is explicitly instructed, often commanded to imitate a posture or action (Jones and Prior, 1985; Rogers et al., 1996; Rinehart et al., 2001; Williams et al., 2001; Noterdaeme et al., 2002; Minshew et al., 2004; Jansiewicz et al., 2006; Mostofsky et al., 2006; Gidley Larson et al., 2008; Gowen et al., 2008; Haswell et al., 2009; Fournier et al., 2010a,b; Izawa et al., 2012).

In contrast to the scarce ASD research regarding the potential roles of SBV in shaping the movement-based kinesthetic percept, an important body of knowledge has accumulated over the years in ASD with a focus on visual and auditory perception and their potential roles in cognitive specialization. Some of these spatial-processing capabilities can rather successfully lead to visuo-spatial or audio-spatial strengths, sometimes paired with complex visualization or auditory abilities (Samson et al., 2011, 2012). This literature examines differences in perceptual processing and over-reliance on complex specializations as successful adaptations of the autistic systems. These could possibly be self-discovered to bypass corrupted sensory input. From the therapeutic standpoint, this observation potentially opens new avenues where we could explore the possibility of sensory-substitution in ASD.

Sensory-substitution is germane to biological systems in general. When some of the sensory input is corrupted or lost in one modality, that missing information can be replaced with sensory input from another modality. Examples abound where a blind person learns to echolocate (Veraart et al., 1992; De Volder et al., 1999), or a person who loses his movement-based proprioception learns to control his body movements using vision (Cole, 1995; Riso, 1999). In all cases where there is cross-sensory transfer (Levy-Tzedek et al., 2012) the sensory-motor systems learn to close the sensory-motor feedback loops, to receive re-afferent sensory input in a compensatory manner that helps regulate the efferent motor output in anticipatory ways.

Anticipatory control of our actions is “the name of the game” in decision making. Decision-making is critical in all intentional aspects of our behaviors. In the words of Henry Markram “Decisions are the key things that support our perceptual bubble, that keep it alive. Without decisions you cannot see, you cannot think, you cannot feel … ” (TED talk, 2009/10/15 http://blog.ted.com/2009/10/15/supercomputing/). In the case of the affected nervous system, by closing the sensory-motor feedback loops the affected individual could regain predictive control of his/her actions and build motor expectations. This would enable the person to anticipate the consequences of immediate future actions and weigh the risks and benefits of impending decisions. That is, the person would regain or develop the ability to be thinking in the abstract, navigating a step ahead of the actual physical act; behaving without necessarily having to experience the physical external input during the action: without having to exclusively rely on “the here and now.”

Given the often reported enhanced visual and auditory processing capabilities of individuals with autism (Mottron and Belleville, 1993, 1995; Mottron et al., 1998, 1999, 2000; Caron et al., 2004; Soulieres et al., 2009; Bonnel et al., 2010; Samson et al., 2012) and their statistical reliance on the “here and now” (Torres et al., 2013 in this issue), we asked if using sensory-substitution to bypass corrupted proprioception with visual and/or auditory feedback could help us connect their intentions to their actions. To this end we used SBV as a proxy to evoke and sharpen intentional behavioral variability (IBV). We then used precise statistical indexes to assess possible gains in volitional control over their own hand motions.

We present a new platform for *personalized intervention* where we close the sensory feedback loops by augmenting the physical external reality of the child with media. In this context we evoke the triggering and regulation of the temporal unfolding of the media using real time motions of their hand. In closed loop with the media, by co-adapting the statistics of his/her own physical micro-movements with those of the media states, the child spontaneously learns. Without instructions, each individual self-discovers where to move the hand to activate and eventually sustain the media.

We show that using this co-adaptive, closed loop interface is ideal to unveil the best form of sensory guidance (e.g., auditory, visual, or touch) that leads an individual toward a more predictive regime of behaviors. In this context the media-states' statistics and the statistics of the child's hand motions are interchangeably used as feedback to modify future performance. The use of our new SPBA enables us to dynamically track the rates of change of the hand-motions' stochastic signatures as the child explores and—through trial and error—self discovers the implicit goal of the task and solves it. We describe using precise statistical indexes how this general statistics driven co-adaptation concept, using sensory-substitution to close the sensory feedback loops, can lead to the development, retention, and improvement of intentional self-autonomy.

## Methods and apparatus

### Motor variability revisited: a necessary preamble to our methods

Motor variability has come to play a relevant role in contemporary movement research, from infant development to adult performance. Inspired by the pioneering works of Esther Thelen (Thelen and Fisher, 1983a,b; Thelen and Smith, 1994) and Nikolai Bernstein (Bernstein, 1967) recent work has begun seriously considering behavioral variations and behavioral variability as useful quantitative research tools. An example specifically focusing on infant development can be appreciated in a special issue of Physical Therapy (2010 Volume 9) highlighting the important roles of motor variations and variability in childhood development as well as their potential use in diagnosis of early neurodevelopmental problems (Dusing and Harbourne, 2010; Fetters, 2010; Hadders-Algra, 2010; Vereijken, 2010).

It is important, however, to point out in our present report the fundamental differences between our new statistical approach to motor variability and the traditional approaches currently in use. To better appreciate such differences we quote Helders from the special issue (Helders, 2010): *“Intra-individual variability can be defined as differences in motor development or performance within individuals and between repeated measurements. The term ‘fluctuations’ is reserved for differences among consecutive points in a variable trajectory, whereas ‘stability’ indicates the counterpart of (or the lack of) variability.”*

In the papers of that important special issue many forms of variability are defined, ranging from variations across the repertoire of tasks that an infant may develop to more specific statistical variability within a task. Statistical variability in the context of Dynamic Systems Theory used by these researchers and others, specifically refers to the “*Measure of how variable a specific, defined behavior is around a central value; typically measured using means and standard deviations and related to the amount of range of a movement or behavior*” [see Table 1 Definitions of Key Terminology in Dusing and Harbourne (2010) taken from (Stergiou et al., 2004)].

Our approach using variability as an objective quantitative tool is, in at least two important ways, fundamentally different from the aforementioned approaches. First and foremost, we do not define variability around a central mean value, quantified by the standard deviations from that mean value, taken across repeated trials. This definition would implicitly assume the existence of an underlying (theoretically justified) symmetric distribution. This is a dangerous assumption as normality in data obtained from naturally occurring phenomena is not always warranted (Limpert et al., 2001; Limpert and Stahel, 2011). For this reason, we do not assume a symmetric theoretical distribution and summarize the statistics of our data by the mean and the standard deviation (μ ± σ). Instead, we experimentally estimate the probability distribution governing the stochastic random process that gives rise to different statistical signatures in the movement data along with their rates of change specific to each individual (Torres, 2011, 2012, 2013; Torres et al., 2013).

Our recent research using the stochastic approach to assess the continuous flow of movements has revealed that our motions have non-stationary statistics. The probability distributions (experimentally estimated) governing our motions are highly skewed and the values of their parameters shift over time (even at the time scale of a very few minutes). We have found that the two-parameter continuous Gamma family of probability distributions describes with high confidence the human movement data across a wide range of behaviors (reach-to-grasp, pointing, gait, various sports routines, etc.). The degree of skewness and the reliability of the experimentally estimated probability distributions from the Gamma family undergo a maturation process (Torres et al., 2013), yet they change with context and sensory-guidance type at a rate that is unique to each person.

These recent experimental results suggest that it will be critical to personalize our assessments of behavior in compromised systems. Such systems are continually undergoing adaptive changes that observational inventories or metrics based on averaged quantities from discretely tallied scores could not detect. Besides potential confounds from fatigue and boredom of the observer, compounded at times with personal biases and lack of independent validation, such methods chop up the behavior. Behavior, however, continuously flows. The relevant parameters defining the probability distribution of movement kinematics variables at a given time change with the context of the task. They also change as a function of the sources of sensory guidance and as a function of many other developmental and neurodegenerative factors (Torres et al., 2010, 2011). Such dependencies make our proposed metrics ideal to dynamically and individually track the stochastic signatures of continuous behaviors in real time as well as to assess their longitudinal evolution. We not only use them to aid and identify important deviations from typical development and normal aging (Torres, under review; Torres et al., 2013; Yanovich et al., in press). We can also use them to track the rates of change of the stochastic trajectories of our movement variability during behavioral and drug-based therapies.

Unlike the previously cited literature, our approach does not look at fluctuations as “*differences among consecutive points in a variable trajectory”* (Helders, 2010). Rather, our approach (Torres and Jose, 2012) examines—in the context of stochastic processes—the accumulation of fluctuations over time for any given trajectory parameter as the person naturally moves. We have coined this type of fluctuation on the Gamma plane “*micro-movements*” to distinguish it from averaging a parameter across repeats of an action during some elapsed time period (e.g., the number of trials in an experimental session). Such averages are taken under the theoretical assumption of normality while measuring the standard deviations from the mean (Thelen and Smith, 1994; Stergiou et al., 2004; Helders, 2010).

**Figure 1:**
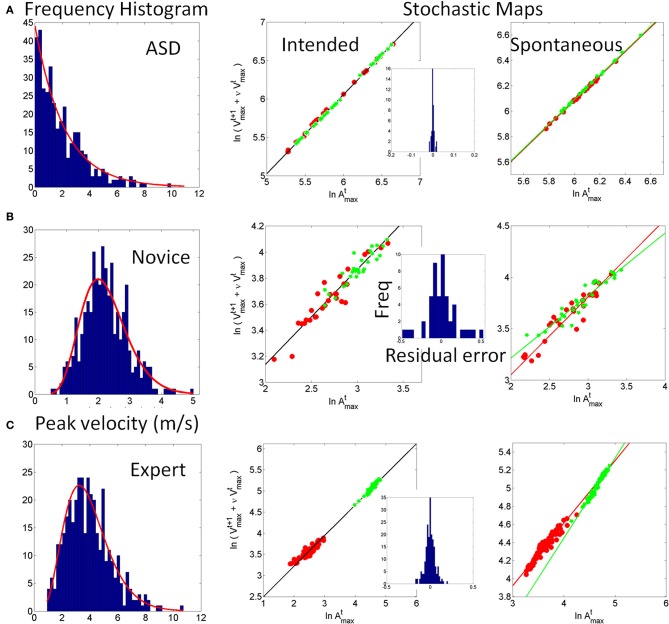
**The concept of motor variability revisited.** An example that lower movement variability in our framework does not necessarily mean better performance. **(A)** Frequency histogram of the peak velocity from JAB boxing strikes performed by an individual with ASD are well-fit by the Exponential distribution (lowest bin centered at 0.16 m/s). Simple speed-acceleration dependent first-order stochastic map used to describe the noise of the performance and to characterize the anticipatory nature (or lack thereof) in speed variability across trials [explained in Torres (2013)]. Each dot represents a trial, red is slow and green is fast. Each speed in trial *n* + 1 depends on the speed and acceleration from trial *n*. The decay parameters of the best fitting relation tend to be well-characterized by a power law and the exponent is generally similar for fast and slow speed when the motion is intended (as in the JAB's strike). The ASD participant had nearly zero noise in the residual error from the fitting and his motions did not group according to speed level as did those from the novice **(B)** and expert **(C)**. In his case the low variability made his motions non-anticipatory of impending speed. Furthermore, the performance was “memoryless” as past speeds did not contribute to the accurate prediction of present speeds and present events did not contribute to the accurate prediction of future speeds. In the panel of spontaneous retractions (reported by the subjects to be beneath their awareness) the ASD performance cannot distinguish speeds and the same slope fits the two scatters (fast and slow) unlike the controls requiring different slopes for a good fit of speed types. In the controls the intended and spontaneous segments of the JAB are different. Here stability is not defined as low variability. The distributions (which are experimentally determined) of the relevant motion parameters are skewed. The experimentally estimated shape and the scale parameters inform of the predictability and reliability of the random process underlying both the intended motion segments and the spontaneous ones that subjects perform without awareness. Rules such as this simple first order one can be informative of anticipatory strategies or lack thereof as the system learns. **(B)** Novice performance (a female spanning lower speed values) and learning the JAB has higher (and evolving) residual error than the participant with ASD whose noise is nearly absent and remains stationary throughout the sessions. Her speeds (randomly called by a computer program during the experiment) begin to group according to speed level unlike those from the participant with ASD. **(C)** Expert performance highly discriminates the randomly called speeds and has low noise (yet his noise level, despite over 20 years of training, is higher than that of the ASD nearly noiseless case). Notice his higher speed levels in the spontaneous segments than in the intended ones along with high discrimination between intended strikes (1 slope) and spontaneous retractions (2 slopes).

Just as the notion of “fluctuation” that our stochastic approach uses is different from that currently in use by others (Stergiou et al., 2004; Dusing and Harbourne, 2010; Fetters, 2010; Hadders-Algra, 2010; Helders, 2010; Vereijken, 2010), the notion of “stability” is also different. In our approach stability of the sensory signal requires high predictability, high reliability and broad bandwidth in the range of values of the motion trajectory parameters of interest. Thus, across repeats of a movement, in our stochastic approach, lower variability in the patterns of velocity and acceleration does not imply higher stability of the system's motor output and motor kinesthetic re-afference. Take for example, Figure 1A from a verbal participant with a diagnosis of ASD who performed a martial arts experiment in our laboratory (Torres, 2011, 2012, 2013). Compare his performance to that of a naïve typical participant in Figure 1B and to that of a typical expert in Figure 1C. According to a stochastic map relating velocity and acceleration maxima in the previous trial to the peak velocity of the impending trial [derived in Torres (2013)] the performance of the participant with ASD is nearly noiseless. This result suggests that his system was not exploring and using the information that is typically present in the natural variability of our actions. The lack of variability in his learning performance was accompanied by the Exponential distribution of his peak velocities. According to the “memoryless” Exponential distribution, past speeds did not contribute to present speeds in any predictive manner. His performance used the information in the “here and now” but did not keep a memory of it that enabled the anticipation of the impending peak velocities from prior velocities in the ways in which the naïve and the expert systems did. In those other systems the fluctuations of these parameters overtime gave rise to informative variability. This in turn led to a stable percept characterized by predictive and reliable statistics (Torres, 2011, 2013). Thus, in our model “stability” does not mean low variability or lack of fluctuations as it does in other approaches to movement variability (Thelen and Smith, 1994; Stergiou et al., 2004; Helders, 2010). On the contrary, movement variability stochastically defined in our approach is the most important part of the learning process. The relevant information lies in the statistical class of variability (rather than in the amounts of fluctuations of the standard deviations around a mean value taken under the assumption of normality). The class of variability reveals the individual rates of acquisition of anticipatory performance and expertise.

These distinctions between traditional approaches and ours are crucial as they open a completely new way of assessing *change* in the continuous flow of natural motions, both in real time and longitudinally. By itself, a micro-movement does not convey any meaningful information. It is the accumulation of these fluctuations over time that informs the system of expectations (or lack thereof). Consequently, in our new approach, the question is not whether a child has less or more variability in his/her motions. It is rather whether the rate of change of the *experimentally estimated parameters* describing the probability distribution underlying his/her motions' fluctuations describe a reliable and predictable random process with broad, explorative bandwidth of values (Torres et al., 2013). Those properties make the sensation of our motions emerge as a stable, predictive, verifiable, and anticipatory percept in a very precise statistical sense. Furthermore, in the context of the degrees of freedom (DoF) problem posed by Bernstein (Bernstein, 1967), we can assess stochastic patterns of variability along the dimensions of the space of body configurations that are relevant to the task at hand. These stand in contrast to the spontaneous variability of task-incidental dimensions (Torres and Zipser, 2002, 2004; Torres et al., 2011) so as to precisely examine in real-time the balance or lack thereof between the voluntary and spontaneous aspects of complex behaviors where multiple DoF continuously interact.

There is a second crucial difference between averaging across repeats of an action within a point-to-point given segment and tracking the stochastic signatures of micro-movements over time at the motor output. The former conceives movement as averaged efferent information within some elapsed time without informing us about non-stationary shifts of the efferent motor execution output in real time. This information, which we continuously track in our stochastic approach, is critical to gain a handle on the proprioceptive sensing of our real-time continuous flow of motions as re-afferent input, possibly sensed by kinesthetic transducers. Our definition of this type of sensory input can provide a precise metric of the emergence of a stable and reliable motor expectation (percept). Once that percept turns stable and tractable within the sensory-motor systems, it is also impinged by other forms of sensory guidance including the movement execution itself, all of which bring in new fluctuations.

We provide a way to measure the statistical anchors that the system self discovers in the “kinesthetic priors” that it builds and constantly adapts. This information contains a bundle of intermixed sensory and motor inputs. In the near future we need to deconstruct this bundle and develop new methods to separate various external from various internal influences. Yet at present, using this new personalized statistical platform, we can already track in real time the fluctuations and the acquired stability of this movement-based continuously flowing information on an individual basis. We can do so within the stochastic feedback control framework that others had previously introduced to the field of computational motor control (Todorov, 2005, 2009) but for which, up to now, no experimental estimation of the probability distributions underlying our continuous, unconstrained, natural behaviors had been provided during development and/or adulthood.

Esther Thelen proposed “… movement must itself be considered a perceptual system” (Thelen and Smith, 1994), p. 193. However, up to now no proper statistical framework had been suggested to provide a working definition to test this important proposition. Such a framework would have to enable the real-time and/or longitudinal tracking of the evolution of movement as a form of sensory input during infancy and adulthood. It would have to enable the assessment of the maturation process of our movement sensation as our sensory-motor systems learn to stabilize that sensation, turn it into a reliable signal and adapt that percept throughout our lives.

The new, stochastic notion of movement-based kinesthetic re-afference proposed by our group (Torres et al., 2013), the experimental assessment of motor-based kinesthetic sensations along with their emergence as a stable, reliable, and diversified percept permit the tracking of our continuous flow of movements over time as a form of sensory feedback. In our approach this information is tracked in tandem with basic cognitive processes involving decision-making and anticipatory estimation of the consequences of our actions (Torres, 2013; Torres et al., 2013).

For all the above mentioned reasons, the assessment of this form of proprioception and sensory feedback in our approach is radically different from what had been done in motor-related research in autism up to now, e.g., (Gidley Larson et al., 2008; Haswell et al., 2009; Izawa et al., 2012). Prior work had not provided a way to close the sensory-motor feedback loops and to quantify the continuous exchanges between decisions and actions *in real time*. They gave us a static snapshot of the system disconnected from basic cognitive decision making, and devoid of SBV. For a more in-depth review of the ASD motor literature we refer the reader to the introduction of Torres et al. (2013). In the present report we rather focus on the application of the new statistical framework to track kinesthetic motor re-afference in real-time and longitudinally within a new experimental therapeutic intervention concept.

### Participants

A group of 25 individuals with ASD participated in this study, (17 males and 8 females) as well as 8 TD controls (6 males and 2 females). The ages ranged from 6–25 years of age for the individuals with ASD and 3–5 years of age for the TD controls. The IQ score of the individuals with ASD ranged between 40 and 107. Demographic information is presented in Tables A1, A2. Parents signed parental consent for the children and young adults provided their consent. The protocol was approved by the Institutional Review Boards at Rutgers University and at Indiana University in compliance with the Declaration of Helsinki.

### Setup

We designed an experimental setup that encourages intentional exploration of 3D space. The subjects were seated in front of a computer screen at a distance that often prevented them from touching the screen but that it encouraged them to point at the screen. They were wearing electromagnetic sensors (Polhemus Liberty, 240 Hz) attached to a vest and secured with Velcro strips to their hand, forearm, upper arm, and shoulder. The sensors and attaching Velcro were embedded in customs with different Disney themes of the children's liking. This assisted us in the processes of setting up and speeding up calibration.

**Figure 2:**
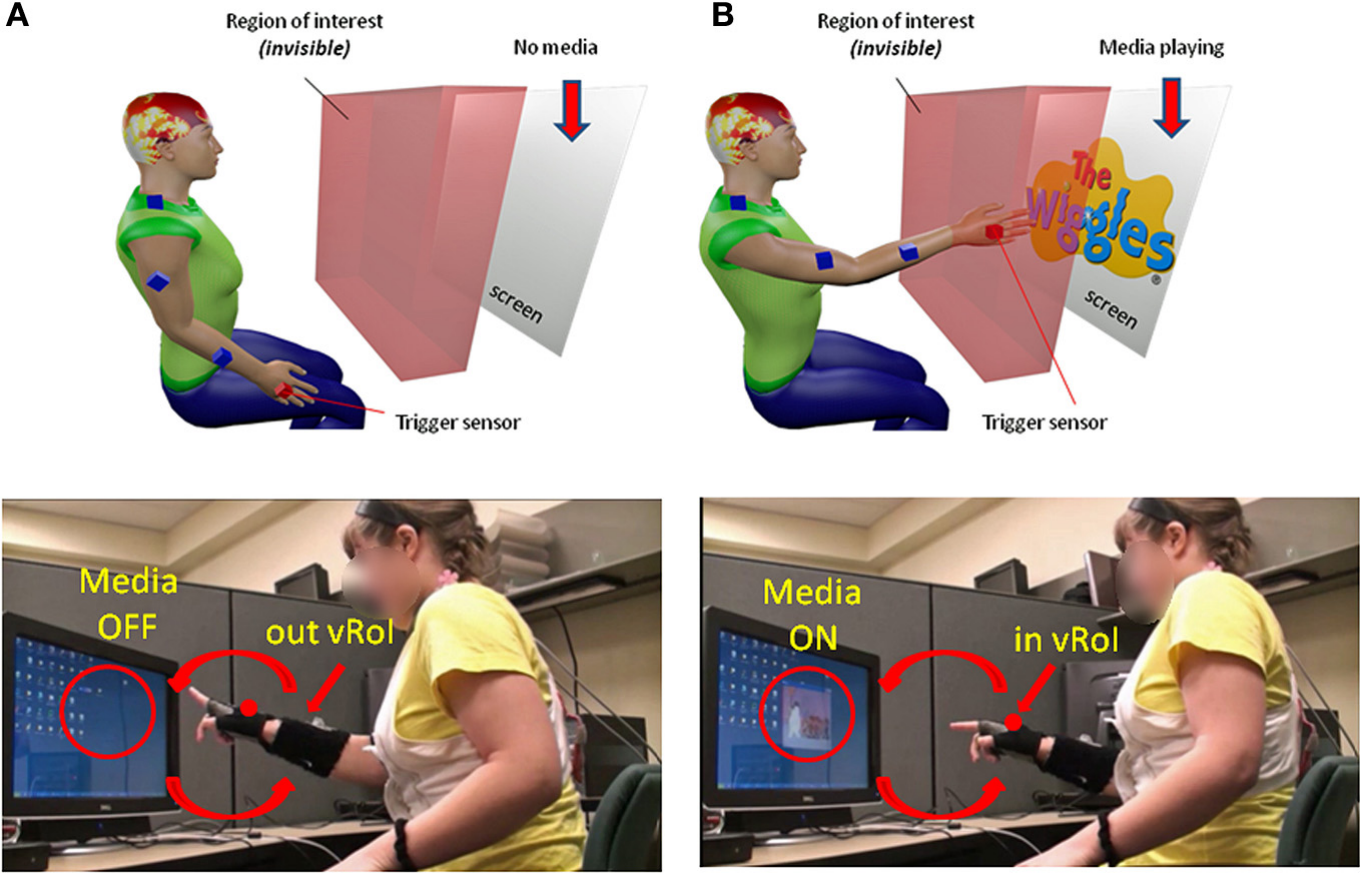
**Experimental Therapeutic Intervention: closing the noisy feedback loops by augmenting physical reality with external media.** Co-adaptive, closed-feedback loop interface connecting the real time spontaneous movements of the hand and the audio-visual media through cause and effect so as to evoke goal-directed motions. **(A)** The set up consists of the person, media, and a way to capture the physical motions of the person's hand (electro-magnetic sensors in this case sampling at 240 Hz can also be replaced by video cameras). A virtual region of interest (vRoI) invisible and unknown to the subject is created in the peripersonal space. **(B)** Movements of the hand that cross that region will trigger the media ON and provide instantaneous explicit audio-visual feedback to the subject about his/her hand motions causing that effect. The vRoI can be a volume, a plane, a small area, or a grid of points. It can be moved around during the session or it can remain static in one place as in the present example. The child receives no instructions about the goal of the task or the way to accomplish the goal. She/he comes to uncover the goal which is to deliberately sustain the hand inside the vRoI in order to continuously play the media. Movements are registered and the shifts in their stochastic signatures tracked in real time to determine the media that drives the behavioral variability toward more predictive signatures conducive of anticipatory motor control.

Somewhere between the subject and the computer screen a virtual region of interest (vRoI) was defined by the experimenter, which the subject could not see (Figure 2A). This vRoI could be moved around and flexibly defined by the researcher as a local square, as a plane or as a 3D-volume of variable size. In this work we used a plane with volume. The goal of the task was implicitly designed so that the subject had to self-discover it. The implicit goal was to hold the hand inside the vRoI so as to trigger and continuously play external media. The subject could use both hands to explore the space but in this report we focus on the use of one hand at a time to transiently trigger the external media when crossing the vRoI and sustaining the media playing when holding the hand inside the vRoi. That is, only real time movements from one hand were tracked so as to register the entrance into the vRoI, the exit from it and the time period when the child was steadily keeping the hand inside the vRoI (see movie at http://www.youtube.com/watch?v=2DKc6aSgd20&feature=youtu.be). The external media could be:

1. Real-time video of the participant facing the monitor captured using a built-in camera facing the participant.
2. Cartoons with sounds (music, dialogues, etc.) of the participant's interest.
3. Cartoons with sounds (music, dialogues, etc.) that were not of the particular participant's interest.

The experimenter in coordination with the educators and therapists of the school (the Douglass Developmental Disability Center of Rutgers University, DDDC) compiled for each child the list of preferred and non-preferred media before the experiments began.

As the participant moved the hand around his/her peripersonal space the hand's changes in position and orientation in the 3D-space were tracked in real time so a computer interface could automatically detect entrance to and exit from the vRoI. This was based on the Euclidean metric tracking in real time the distances from the current hand position and orientation to the current position and orientation of the vRoI defined by the experimenter. Whenever the hand entered the vRoI (distance close to 0 with tolerance error set by the experimenter) the media was automatically triggered by the interface. If the hand remained at that spot, the video would continuously play (Figure 2B). If the hand moved out of the vRoI, the video would stop (Figure 2A). The subject had to realize these contingencies on his/her own. The motion was captured continuously and time-stamped as IN or OUT the vRoI. The stochastic patterns of the motion were analyzed using the SPBA that we describe next.

### Sensing movement from the peripheral limbs: limb proprioception-based media selection

We used recording session lengths of 50 s and above. The majority of subjects had 2 or more sessions either the same day or on different dates (several weeks later). The sessions for the same subject involved different types of media which allowed us to evaluate movement-sensing (proprioceptive)-based media preferences. If the media made the motions statistically more predictable (in a very precise sense to be defined below), the media was considered as preferred. If the media made the motion patterns more random and noisier, the media was considered as non-preferred. The time-scales of these progressions were also automatically recorded to validate the notion of preference.

### Parameters of interest

**Figure 3:**
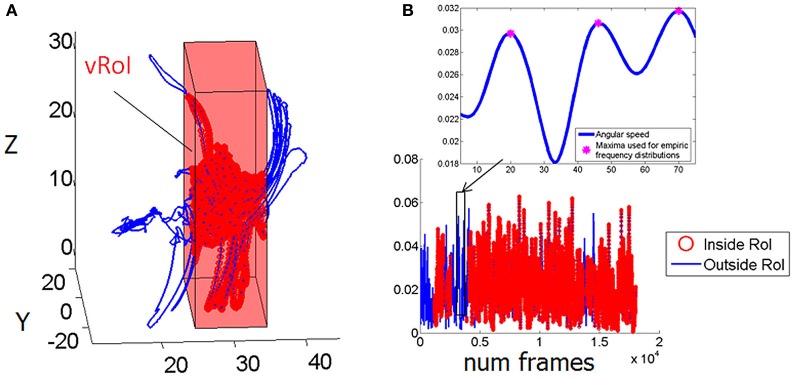
**Velocity dependent micro-rotations of the hand registered in real time while exploring the peripersonal space. (A)** Virtual Region of Interest (vRoI) invisible to the subject marked as a cube colored in red. Hand positional trajectories captured at 240 Hz inside and outside the vRoI as the child continuously explores the space during 83.3 s. **(B)** Hand rotations displayed as angular speed (radians) obtained by parameterizing the rotations using the quaternion representation and computing the (Euclidean) norm of the angular velocity vector. Blue marks the rotations of the hand outside the vRoI and red marks them inside the vRoI. The peak angular velocity (marked by the magenta star) and the time (ms) to attain it from each minimum are the parameters of interest to track their micro-movements.

We collected 3D position (Figure 3A) and orientation (Figure 3B) from all four sensors at the rate of 240 Hz as well as the hand sensor status (Figures 3A,B, red IN, blue OUT), which was based on the distance between the moving sensor at the hand and the plane defining the vRoI.

#### The velocity-dependent hand kinematics

The angular speed of the hand sensor was obtained from the changes in hand orientation tracked at 240 Hz. We parameterized these rotations using quaternions (a vector of four dimensions to represent points in the special group of rotations; Kuipers, 1999). A quaternion is a vector of 4 numbers. Three of them specify the unitary vector (axis) of rotation in 3D space and the fourth is the angular magnitude of the rotation of the rigid body (the sensor attached to the hand) around this vector. We defined angular speed as the Euclidean norm (the square root of the sum of squares, taken component wise) of the vector consisting of these 4 numbers. An example of the angular speed continuous sequence is shown in Figure 3B for one session and media type; the inset box zooms in the long sequence for just a few frames to show the parameters of interest.

The main advantage of using the angular velocity is that it quantifies the changes in the hand posture (independent of the parameterization of the rotations). Moreover, the outcome of the rotation was neither instructed, nor restricted by the size and location of the region of interest (a goal of the task which the participant had to ultimately discover).

### Statistical platform for personalized behavioral analyses (SPBA)

We estimate the probability distribution best characterizing the experimental frequency distribution of the hand's trajectories as it continuously crossed from the OUT to the IN vRoI. We use in this case angular velocity (Figure 3B) and read in a minimum of 100 points per estimation. The time scale of these readings will depend on the sampling resolution at the researcher's disposal. However, the rate of change of the stochastic trajectories generated by the person will be independent of this so long as the number of readings is large enough to have proper estimation with adequate goodness of fit tolerance values. For example the MATLAB algorithms for maximum likelihood estimation (MLE) of the parameters of the probability distributions will output the 95% confidence intervals for each estimated parameters and the goodness of fit values. In our case since the sampling resolution is 240 Hz we can obtain 100 readings of peak angular velocities in a few seconds and over minutes, sample densely the rotational motions of the wrist joint angles.

As previously explained by using the stochastic approach, we treat these fluctuations in the joint rotations as re-afferent sensory feedback continuously flowing between the peripheral and the central nervous system. As the actions continuously unfold, these micro-movements are sensed kinesthetically from the physical motions by joint and skin receptors and by muscle spindles.

**Figure 4:**
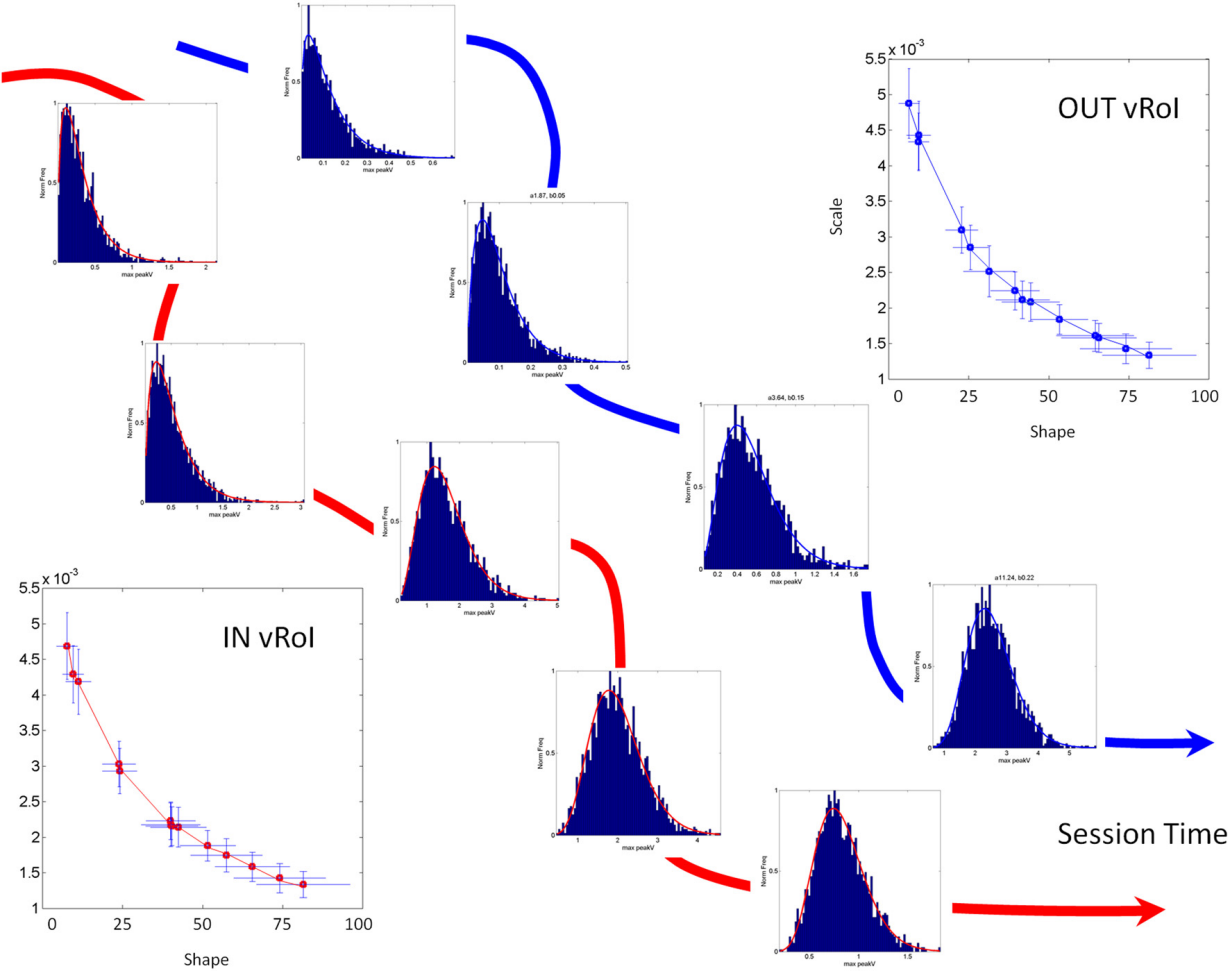
**Schematic explanation of the statistical platform for personalized behavioral analyses (SPBA) tracking the non-stationary statistics of continuous movements in real time.** (The data sets in this schematic drawing were synthetically generated for explanation purposes using the actual Gamma estimates from a child's behavior as the seeds). The fluctuations in the peak angular velocity of the sensor at the hand are accumulated as the wrist rotates while the child explores the peripersonal space with his/her hand. The fluctuations occurring IN vRoI (red) are accumulated in parallel to those occurring OUT vRoI (blue) in the continuous flow of motions of the hand's behavior. Over time the behavior transitions from random to exploratory to systematic and goal directed, to intentional (as when the child deliberately holds the hand to sustain the media playing). In any given time period there can also be reversals toward a noisier random pattern as a function of the media. The faster the convergence toward anticipatory behavior (predictable, reliable and exploratory), the more preferred the media is by that child. Lower media preferences would thus be accompanied by stagnation in this process and reversals toward noisy and random patterns (up and to the left of the Gamma plane). The normalized frequencies histograms of the peak angular velocity represent selected times across one session as the stochastic trajectories IN and OUT vRoI evolve in time. The histograms are fit with members of the two parameter continuous Gamma family of probability distributions. The estimated (shape, scale) parameters are plotted as points in the Gamma plane with 95% confidence intervals. The stochastic trajectories from the orderly shifts are represented on the Gamma plane. Points to the right and down are predictive and reliable, thus marking anticipatory behavior. Points to the left and up are random and noisy. Notice that there are differences in the rate of change of the stochastic signatures. Underlying curves represent the flow of time in the session.

The two parameters of the continuous Gamma probability distribution family, the shape and the scale, can be estimated from the experimental data using MLE algorithms and plotted in the Gamma (*a,b*)-plane (Figure 4) with 95% confidence intervals to label each individual during the baseline state, early in the task. As the system interacts with the statistics of the environment, the statistics of the continuous flow of hand motions change. These shifts can be captured over time as (*a,b*) points of the Gamma (shape, scale) plane, which span a trajectory (Figure 4). This trajectory will have different rates of change in direction and magnitude, which we can track as well. The latter uniquely define the person's compliance with or resistance to the manipulation of the sensory input that we use. In the Figure 4 we provide an example in schematic form of the parallel learning process as the hand explores and moves IN and OUT of the vRoI.

We accumulate the minute fluctuations (micro-movements) on the Gamma plane and build a stochastic trajectory from the statistics of the hand's maximal angular speed, as the hand repeatedly enters or leaves the vRoI. In Figure 4 we show in schematic form the stochastic trajectories in the Gamma plane for IN vRoI (red) and OUT vRoI (blue). The method applied to continuous human data has demonstrated that the continuous flow of movements in our behaviors has non-stationary statistics (Torres, 2013; Torres et al., 2013). The experimentally estimated parameters of the Gamma family of probability distributions shift values over time with the impinging external and internal sensory stimuli. This feature enables us to dynamically track the stochastic shifts in the Gamma plane in each person and for each individual assess the rates of change of the Gamma parameters caused by the impinging stimuli. This approach helps us establish a causal relationship between sensory input and motor output as it is the system itself that in the closed efferent-re-afferent loop controls the outcome. Thus, we can precisely parameterize the sensory input and readout in the motor output fluctuations the shifts in the stochastic signatures that the parameterized manipulation most likely will cause. Then we can use this feedback in effective ways to accelerate the learning progression toward anticipatory autonomous behaviors. More importantly, we can track these ***rates of change*** in the stochastic trajectories unique to each individual. They can inform us of the maximal shifts toward reliable and predictive signatures and can reveal the best source(s) of sensory guidance: e.g., the source(s) that will most likely turn decisions accurate and fast (Torres et al., 2013).

### Assessing the *continuous* natural flow of behavior

In the present work, since minimal to no instructions were given, the child's system had to learn both to self-discover the goal and find the solution to the “self-discovered” problem in order to get a reward (the triggering of the media) and eventually learn to sustain the continuous media-playing to maximize the reward time. The latter required deliberately holding the hand inside the vRoI once the target area was discovered and systematically visited.

The progression of building such expectations would evolve as follows:

1. Random motions of the hand transiently triggering the media ON and then OFF by chance;
2. Noticing external change in the media state(initially a flash of media);
3. Associating external change in media status to hand motion and space region;
4. Systematically exploring the peripersonal space in search of the “magic spot,” the vRoI which would trigger the media;
5. Measurable shifts in the velocity-dependent stochastic patterns of the hand;
6. Development of intentional motions to keep the hand within the vRoI;
7. Deliberately holding the hand inside the region of interest to sustain the media playing continuously.

For the dynamic tracking analysis we register and separately analyze the periods inside the vRoI from the periods outside the vRoI (Figures 3, 4) for each session. We then take the maximal angular speeds (Figure 3B—zoomed in—inset) that are greater than 0.001 units as the significant rotation cut-off. Since we have participants from a variety of ages and body sizes we normalize these maxima to avoid allometric effects [i.e., we divide the peak angular velocity by the sum of the peak angular velocity and the averaged angular velocity in each rotation: this normalization is typically used in anthropological data (Mosimann, 1970; Lleonart et al., 2000)]. The empirical frequency histograms of the normalized angular speed maxima are then obtained for at least 100 points (as explained above the goodness of fit and 95% confidence intervals were adequate at 240 Hz) for the IN vRoI and the OUT vRoI. In each case (IN and OUT) the shape and the scale parameters (*a,b*) of the Gamma probability distribution are estimated with 95% confidence using MLE. The trajectory is tracked on the Gamma plane as suggested in schematic form on Figure 4. In the present report the points of this trajectory correspond to one recording session. And also, longitudinal assessment was done 2–4 weeks later during a different session with no training in between sessions.

### Quantifying predictability

The Gamma probability distribution describes a continuous family of skewed probability distributions smoothly ranging from Exponential to Gaussian.

The probability density of the Gamma distribution is governed by

(1) $$y=f\left(x|a,b\right)=\frac{1}{b^{a}\Gamma \left(a\right)}x^{a-1}e^{-\frac{x}{b}}$$

where *a* is the shape of the Gamma and governs the degree of symmetry of the distribution; *b* is the scale and governs the height of the distribution. The Γ represents the Gamma function. The larger values of *a* (shape) correspond to more symmetric, therefore, closer to Gaussian distributions. The Exponential case is when *a* = 1.

The Exponential distribution is the only continuous memoryless distribution, whereby previous events do not contribute to the prediction of future events any more than current events do; while the Gaussian distribution has good predictive properties. Thus, a participant whose estimated probability distribution is a member of the Gamma family closer to the Gaussian range, s/he will have motions with more systematic (predictive) behavior than if the estimated probability distribution is closer to the Exponential range of the Gamma plane. Figure 4 shows instances of (*a,b*) estimates corresponding to probability distribution curves, which fit the histograms in 4 with 95% confidence.

In our case the predictive ability (larger shape value) of a certain Gamma family member corresponds to the level of systematicity in the hand posture (orientation) changes. Figure 4 illustrates the shift down and to the right along the trajectory. In general these fluctuations in the Gamma plane can vary in different directions and magnitude but for illustrative purposes they are shown in Figure 4 as the ideal target stochastic behavior that one should aim for if predictability and reliability (low dispersion) are desired.

Our objective was indeed to achieve more predictive regimes once inside the vRoI by aiming for a shift in the (*a,b*) parameters toward the right, to the Gaussian limits of the Gamma plane. The media can be selected according to this objective so as to reinforce the predictive behavioral path to build a reliable motor expectation (a motor-kinesthetic prior) which can result in anticipatory behavior. Likewise, random and noisy statistical regimes shall be discouraged, so the external sensory input (media) that leads to such corrupted proprioceptive signal (toward the Exponential ranges) shall be downplayed.

### Quantifying reliability

The Fano Factor (Fano, 1947) is given by the noise to signal ratio. This is the dispersion of the experimentally estimated distribution, obtained by dividing the estimated variance by the estimated mean. This index can also be obtained for the estimated rates of change of the shifts in the non-stationary statistics of the behavior. In the case of the Gamma probability distribution, the mean is *a* × *b* and the variance is *a* × *b*^2^. This ties the scale *b* parameter to the dispersion because the Fano Factor = variance/mean = *b*. Thus, when the fitting is good these two estimated Gamma parameters provide information about the *predictability* and the *reliability* of the continuous flow of behavior—which we treat as a stochastic process. We specifically aim at systematically shifting the parameter values of the real-time estimated probability distribution *down and to the right of the Gamma plane*.

Using this framework and the new closed-loop co-adaptive paradigm we seek to:

1. Dynamically track the real time evolution of the non-stationary statistics of the velocity-dependent variability as the participants develop predictive statistics and transition from random to systematic to goal-directed, to intentional behaviors.
2. Longitudinally assess the retention of the changes in stochastic signatures: are these changes transient or are they retained and improved when presented with the same stimulus?
3. We seek to automatically and objectively extract from the statistics of the physical movements which media type makes the child's hand motions more predictive so as to reinforce that media type. Likewise we seek to determine which media type makes the hand movements noisier and more random, so as to discount it.

### Important distinctions from current behavioral therapies

This is an estimation process that experimentally obtains the statistical parameters from the behavior—as opposed to assuming a theoretical probability distribution such as the Gaussian distribution and summarizing the process by the mean and the variance parameters, μ ± σ^2^. In current behavioral approaches these measurements are discretely rather than continuously obtained by tallying the *observed* responses over a certain number of trials and obtaining averages. It would be a mistake to do this. The behavior follows a continuous stream. Moreover, the frequency distributions of the kinematics motor parameters underlying the (observationally reported) behaviors have actually been experimentally determined. They do not follow a symmetric distribution. Their frequency distributions are skewed (Torres, 2011, 2012, 2013). It is known that under those statistical features it is incorrect to use the theoretical assumptions μ ± σ or to use parametric models (such as Analyses of Variance, ANOVA, regression, etc.; Limpert et al., 2001; Limpert and Stahel, 2011). Current behavioral approaches do not consider these issues because physical movements present in all behaviors are not currently objectively registered and quantified (Cooper et al., 1987).

This approach is also different than reinforcing the movement itself so as to maximize the likelihood that a particular movement occurs in the future. Current behavioral therapies *command* the child to perform certain movement types. Such therapies try to reinforce a particular movement through repetitions, for example at different speeds, with different stimuli, etc. or to discourage the movement type corresponding to some stimulus set and so forth (Black et al., 1972; Cooper et al., 1987). These actions are driven by the therapist's opinion from tallying the discretized performance by some coding system. This is as opposed to other alternatives such as assessing the stochasticity of the continuous flow of motions—as we do here; or using the fractal dynamics of our motions (Hausdorff et al., 1999) and their metrics of stability [reviewed by Vereijken (2010)]; or using other non-invasive computational techniques to objectively quantify natural performance. Because we want to understand how the autistic system is coping with the corrupted sensory-motor feedback, we do not want to impose any biases in the assessments of their natural flow of movements. We do not seek to reinforce any movement type. Instead we let the child self-discover movement preferences.

Through the stochastic approach we can tell whether or not the motions are more reliable using the Fano Factor, the scale parameter in the case of the Gamma probability distribution. These indexes of predictability and reliability can be applied to any movement. We do not need to enforce or command any particular movement type (as it is routinely done in current behavioral therapies). If we were to enforce a movement type, such commanding would most likely interfere with the spontaneous self-discovery process that we are trying to evoke with our new approach.

The role of the experimenter in this proposed new concept is less active than in current approaches. That is, when following up each individual—through automatic computational tracking—as the system manifests real-time shifts in the stochastic signatures, the experimenter should not interfere with the self-discovery process. The child should lead.

This type of philosophy differs from that of the current behavioral therapies [e.g., Applied Behavioral Analyses (ABA) http://www.youtube.com/watch?v=SLBLnNxzftM], where the therapist would be the one determining which stimuli/behavior/letter would be the best to reinforce in any given session based on observation of the responses of the child. By objectively quantifying the continuous flow of expected behaviors in closed loop with actual physical behaviors driven by the child it will be possible to get at the implicit aspects of the learning process that the human eye will inevitably be missing when exclusively focusing on the discrete goal-directed segments embedded in the continuous flow of behavior.

The more traditional form of feedback-based correction tends to be rather centered on the experimenter's inferences. Yet, we aim here at shifting that focus from the experimenter's inferences to the statistical inferences based on the physical motor outcome of the child. Under these self-driven actions, we aim at using the SBV inherently present in the child's micro-movements as a proxy to spontaneously evoke intentionality in the child's actions. Intentionality in this case goes above and beyond attaining goal-directness. The objective is rather to have the child's self-discovered behaviors evolve toward statistical patterns with stochastic signatures that have precisely defined predictability and reliability ranges according to the statistical indexes (as defined above).

In the present experimental therapeutic intervention the statistics of the child's movements (rather than the experimenter's opinion) reveal the best source of sensory guidance: i.e., the media type that maximally shifts the probability distributions toward reliable and predictable regimes. This is done automatically, independent of the experimenter's inferences. It is through exploration and self-discovery that the child comes to find out what the problem is; solves it; and obtains the reward. The reward in this case is not food or a token, but the very solution of the problem: the media continuously playing, thus making the child acquire volitional control of his/her movements. This direct cause-effect realization and active use of intentionality by the child alone is at the core of this proposed intervention concept and the rewarding control of the child's own actions.

The non-stationary statistics of the natural flow of movements during the exploratory behavior and the analyses of the indexes of performance (reliability and predictability) permit real-time rapid and automatic personalized assessment of the child's preferences of self-video *vs*. cartoons, movies, music, dialogs, etc.

### Times IN/OUT of the virtual region of interest

To establish the signatures of variability for motions IN and OUT of the vRoI indicating media preference we examined the time spent in random motions (or in goal-seeking) OUT of the vRoI; as well as the time spent goal-contacting IN the vRoI. The time in this case corresponds to the number of frames registered at 240 Hz resolution (which divided by 240 provides the number of seconds spanned by the frequency of visits to one region of space or another). Given a media type, the systematically higher % session time spent IN the vRoI simultaneously combined with faster rates of shift in stochastic signatures toward the more predictive and reliable statistical regimes of motion variability are the criteria for “preferred media.”

**Figure 5:**
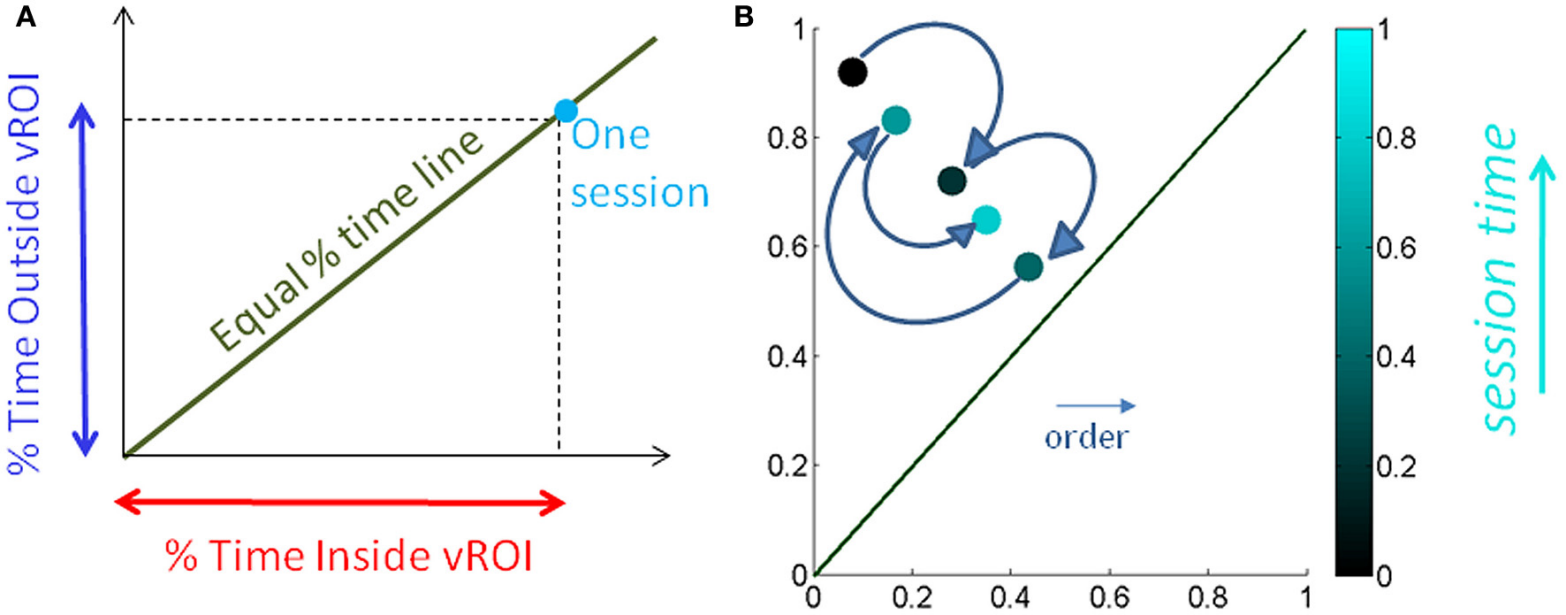
**Media preference metric validation for a session. (A)** We used the percentage of time spent inside the vRoI *vs*. percent of time spent outside the vRoI to validate that more time inside the vRoI (lower right area, under the green line denoting equal percentage of time spent inside and outside vRoI) correspond to subject's stochastic signatures being in more predictive range. **(B)** We also looked at the time percentage inside the vRoI progression as the recording session goes on in a window of 20% of session time, to get a feeling of dynamics of the interest taken by the subject in media. As the recording session goes on, the colors get brighter. The ideal exploratory pattern would be when the dots shift toward lower right as the colors would become brighter.

We normalize the times by the session length to avoid the effect of varying session time lengths (Figure 5A) and express this parameter as a % of time. We also look at the sliding window progression of the fraction of time spent inside the region of interest within one session to get a sense of the exploratory progression within that session. We chose the sliding window size as 20% of session duration, as this was the minimum ideal window across all session lengths. And we can track, combining the Gamma parameter estimation and the temporal metric, the precise temporal progression of the stochastic evolution as a function of session time. The temporal metric for a session is depicted in Figure 5B where the session time is color-coded from darker (earlier in the session) to lighter (later in the session). The arrows highlight the order of the trajectory.

## Results and discussion

### The two-parameter continuous gamma family of probability distributions captures both TD and ASD behavioral variability

**Figure 6:**
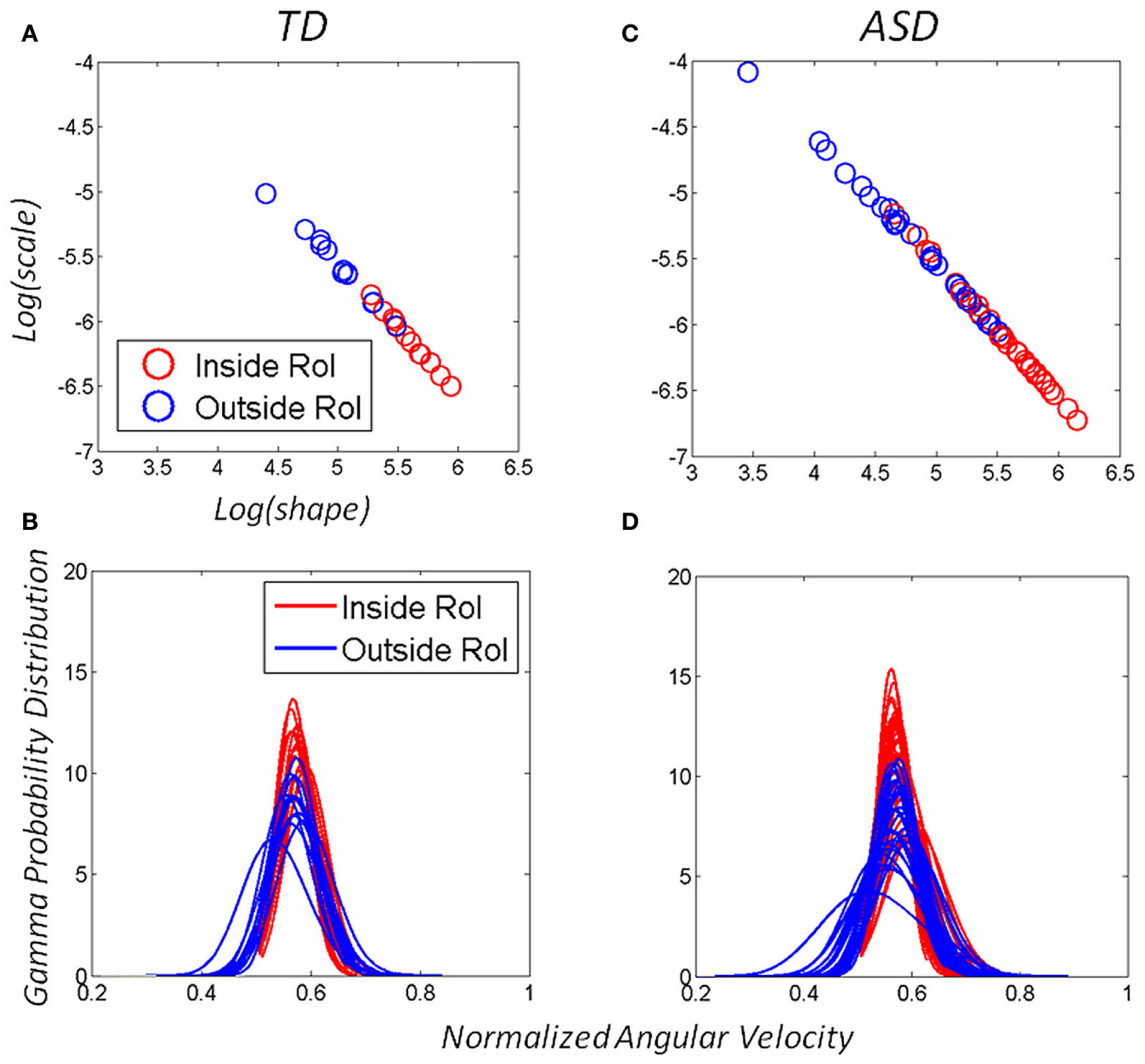
**Adaptive capabilities of participants with ASD: both TD children and children with ASD shifted the stochastic signatures of the angular-velocity dependent micro-movements.** The orientations of their hand became more predictive as the hand deliberately remained in the vRoI to continuously play the external media. The estimated Gamma parameters for the normalized angular velocity [peak angular velocity/(peak angular velocity + averaged angular velocity)]. In this case a sliding window was used to track the stochastic value of the parameter every 100 peak velocity values as the hand explored the space in search for the vRoI and as it discovered it and the hand was held there continuously to sustain the media playing. **(A)** Patterns of a TD child. **(C)** Patterns of a child with ASD. **(B–D)** The Gamma probability distributions were estimated from the empirical data within the range of normalized angular velocity values for each child. Note that the child with ASD starts out with higher dispersion in the distribution (variance to mean ratio) but as the vRoI is discovered and the hand sustained inside it, the noise-to-signal ratio decreases, thus increasing the reliability of the probability distribution.

The distributional analyses revealed that the Gamma family captured with high confidence the stochastic signatures and their shifts for each one of the TD and ASD participants. Each child's hand angular velocity peak inside the vRoI as well as outside the vRoI spanned a frequency histogram well-fit by one of probability distribution members of the Gamma family. The shape and scale parameters were plotted and tracked in the Gamma plane as they evolved with the search for the vRoI and the media type. Figures 6A,C show the scatters on the Gamma plane for each set of participants. Figures 6B,D show the estimated probability distributions of the normalized peak angular velocity corresponding to (**A** and **C**).

The evolution of the parameters revealed shifts in the stochastic signatures of each child with different step size and different directions (different rates of change). Shifts in the Gamma plane were sometimes to the left thus indicating a change in the shape of the distribution and more randomness in the variability. Other shifts were to the right indicating a change in the shape of the distribution to a more symmetric type, thus signaling acquired predictability in the variability. Likewise shifts up and down along the scale parameter helped determine the degree of dispersion in the distribution and informed of the reliability of the underlying random process. We report the values of the estimated parameters and the goodness of fit for each group on Tables A2, A3.

### Motions outside the vRoI were less predictive than those inside the vRoI

We focused on two types of motions within the continuous flow of movements that the Gamma signatures revealed. The motions OUT vRoI turned out to be more random as their signatures were more often to the left of the Gamma plane than those of the IN vRoI cases. As the child's search became more systematic outside the vRoI, these motions also shifted the stochastic signatures to the right of the Gamma plane with consistency. Figures 6A,B show this trend in both the TD and the ASD groups.

### The rate of change of stochastic shifts and the temporal metric reveal the media preferred by each child

**Figure 7:**
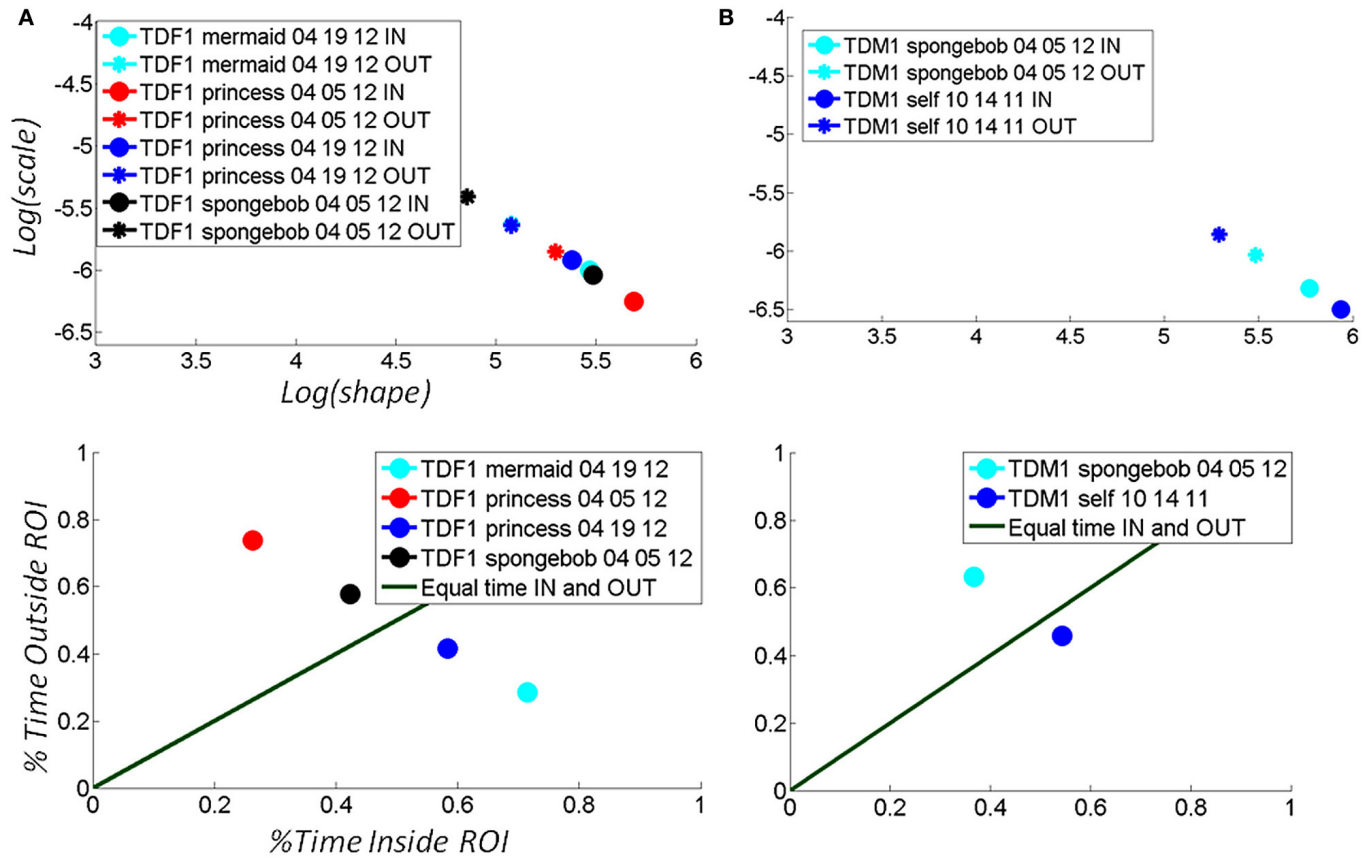
**Selectively finding and using the media type that shifts the stochastic signatures of the micro-movements of the TD child's hand toward the more predictive regime: building behavior that shifts the statistics of micro-movements toward spontaneous autonomous control. (A)** The hand motions of a TD female were tracked longitudinally over the course of a week, shifting toward a region of more statistically predictive behavior as the hand spent more time in the vRoI (from red to black) when triggering a Disney cartoon involving “The Disney Princesses”. Bottom panel shows the percent time spent inside (systematically exploring) the vRoI *vs*. that spent outside the vRoI (randomly exploring). This particular TD girl preferred “The Little Mermaid” video over the “SpongeBob SquarePants” video, demonstrated by the stochastic patterns of angular velocity micro-movements shifting to more systematic regimes for the former rather than the latter. Likewise, we longitudinally quantified improvements over the course of a week using the preferred media (from red to cyan). **(B)** A TD male prefers the real-time video of himself to the “SpongeBob SquarePants” videos. He spends over 50% of the time in the vRoI for the former media, deliberately holding his hand to watch himself, and over 60% of the time outside the vRoI exploring during SpongeBob. In the top panel, the patterns inside the vRoI can be seen to be consistently more predictive as they fall farther to the right of the Gamma plane than those outside the vRoI during random exploration.

For each child the maximal step size of the shift of the stochastic signature given by the change over time of the (*a,b*) position in the Gamma plane was unique. Figure 7 shows the evolution of the stochastic signatures of two TD children. The shift in the (*a,b*) points provide the rate of change of the stochastic signature over time. The maximal size in shift to the right (more predictive behavioral variability) among a set of media reveals the media that causes the shape of the probability distribution to turn more symmetric. Notice here that this is not just a correlation as it is the child who is in real-time, in closed loop with the media, causing the shifts in stochastic signatures from the hand motions to become more predictive.

This indicates that the goal-seeking movement patterns become more predictive and more reliable as the child searches for the “magic spot” and moves the hand outside the vRoI to try and trigger a particular media type. The largest shift in the stochastic signatures down and to the right of the Gamma plane reveals for each child the media type that would most likely maximally accelerate the acquisition of more predictive, reliable, and diversified movement patterns. Such media leads to the self-discovery of the primary implicit goal of the task (i.e., finding the vRoI) and in turn, to systematically accomplish the secondary implicit goal (sustaining the hand in that vRoI to continuously play that media).

These goals and sub-goals are implicit as they are not instructed but must be self-discovered. However, over time they shift priorities so the secondary goal becomes the goal of the task of playing the media continuously by holding the hand in a particular position of space (the vRoI). The relation of the change in distribution shape for each child and the Fano Factor quantifying the dispersion of the distribution is plotted in Figure 11. The Figure 11 shows the worst and the best cases where the predictability and reliability of the INvRoI and the OUTvRoI cases are respectively quantified. Notice that the TD children separate the slopes of the power relations with faster rate of change for the INvRoI cases. Table A2 provides examples of the evolution of the parameter for different media types.

A temporal metric revealing a systematic gain within a session is given by the frequency of the times during that session that the child's hand moved inside the vRoI *vs*. the frequency of the times that the child remained exploring the space outside the vRoI. This is quantified through the percent of time that the hand remained in each region (IN *vs*. OUT) which systematically coincided for each child with more predictive (IN vRoI) stochastic regimens or less predictive (OUT vRoI) stochastic regimes.

Figure 7 shows an example for a TD girl (A) and a TD boy (B) of the above metrics and quantifications. Notice that the patterns evoked by the “spongebob” cartoon shifted the stochastic signatures maximally for the girl (7A top black markers) during the 04-05-12 session as compared to the other videos. This can be appreciated in the shift in location down and toward the right of the Gamma plane from the OUT vRoI (black asterisk) to the IN vRoI (black dot). The step size caused by that media type was larger than that for all the other media played that day. Notice as well that in the same session the red markers representing other media had a smaller shift from OUT to IN the vRoI. Overall in that session the hand was exploring more time outside the vRoI but the media represented by the black marker was already shifting toward a regime closer to spending more time inside the vRoI as the motions's variability became more predictive.

The gain experienced in the predictability of the stochastic signatures during the 04-05-12 session not only transferred 2 weeks later to the session of 04-19-12; it actually improved the gains in the percent of time that the child maintained the hand inside the vRoI (blue dot in bottom panel **A**). This indicates that consistently the “princess” video was preferred over the “spongebob” video in the very precise sense of an increase in the frequency of the visits of the hand to the space inside the vRoI and the shifts toward a more predictive location of the Gamma plane. Finally for the girl the video of the “mermaid” had the largest effect as the hand was deliberately spending more time inside the vRoI than with all other media during the second session.

Similar patterns can be seen for the sample data from a TD boy in Figure 7B. Here the real-time videos of himself triggered by placing the hand inside the vRoI shifted the stochastic patterns maximally (step from blue asterisk to blue dot in 7B top panel is larger than step from cyan markers). Furthermore in the first visit 10-14-11 the child sustained the hand inside the vRoI for a longer % of time than in the second visit 04-05-12 indicating that despite the retained gains in predictability (shift to the right) during the second visit, it was the triggering of the real-time self-video that maintained his interest rather than the “spongebob.” He visited the vRoI more frequently in the first visit (blue dot) than in the second (cyan dot) unambiguously informing us that he prefers video of the self over the cartoon.

**Figure 8:**
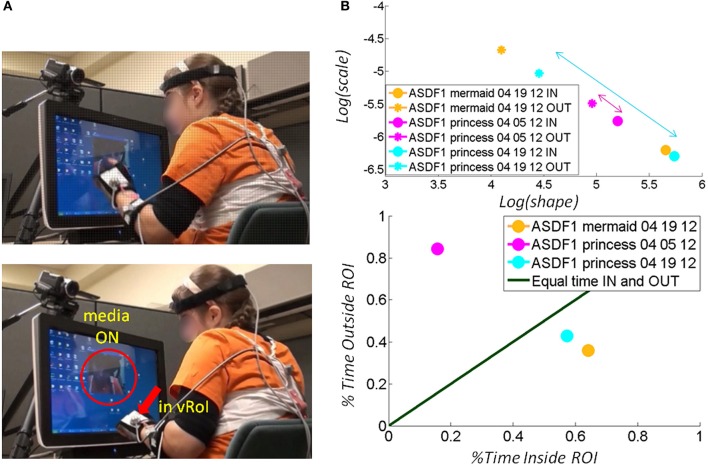
**Tracking the stochastic signatures of angular velocity-dependent hand's micro-movements in a child with ASD. (A)** Non-verbal female child with ASD explores the vRoI and finds out how to trigger and maintain the media ON. **(B)** The real time shifts in the stochastic signatures of her hand movement's velocity-dependent variability are obtained for both the IN and OUT segments of her searching motions. Arrows highlight shifts toward the right (the predictive regimes) of the Gamma plane. The size of the arrow marks the rate of change which is larger (faster) for preferred media. The rate of change of the stochastic signatures speaks of their sensory preferences and adaptive capabilities. Longitudinally (from March to April) there is a shift for “The Disney Princesses” video (from magenta to cyan), but the shift in real time for “The Little Mermaid” is far more pronounced and occurs faster. This video was far more effective than “The Disney Princesses” video in shifting her stochastic patterns toward a more predictive behavioral regime. **(C)** Consistent with the largest shift toward the predictive regimes (preferred media) the child spends more time inside the vRoI with that media type.

**Figure 9:**
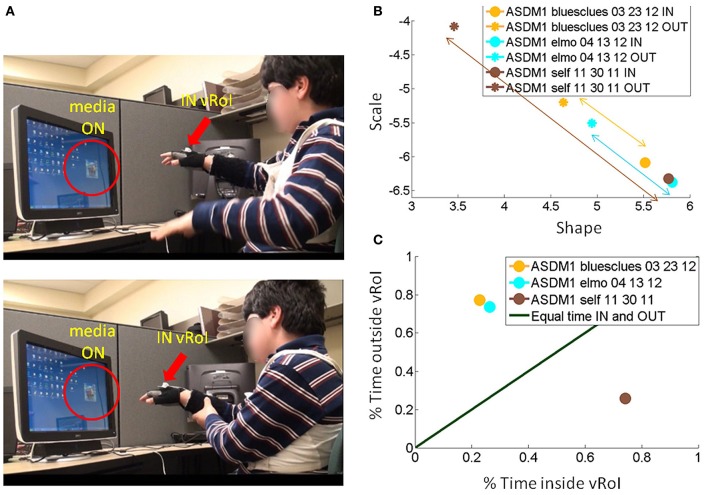
**Tracking the stochastic signatures of angular velocity-dependent hand's micro-movements in a child with ASD. (A)** Non-verbal male child with ASD explores the peripersonal space in search for consistency of the cause and effect phenomena: i.e., the hand triggering the media, and then deliberately holds the hand to continuously play the media once it self-discovers the goal of the task and the solution to attain it. **(B)** Real-time shifts in the rates of change of the stochastic trajectories quantified from the non-stationary statistics of the hand micro-rotations. Largest shifts are due to viewing real time video of himself, while shifts quantified by triggering videos of “Elmo” and “Blue's Clues” are less pronounced. **(C)** The longitudinal measurement of the cartoons show improvements for “Elmo” in both the Gamma plane shifts and in the percent of session time spent exploring the vRoI, yet the percent of time spent deliberately holding the hand inside the vRoI and engaging the real-time video of himself is far larger. Notice as well-that transient predictive gains in 03-23-12 are retained weeks later and improved in 04-13-12 despite no practice of the task in between.

Across ages the participants with ASD also showed adaptive capabilities as the TD children did. Remarkably, the non-verbal participants became as engaged as the TD participants and as their verbal ASD peers. As with the other participants the non-verbal participants with ASD spontaneously figured out the goal of the task and came to a correct solution without instructions. Examples of non-verbal children with ASD are shown in Figure 8 (a girl) and Figure 9 (a boy). Notice that the girl with ASD showed the largest shift for the “mermaid” (Figure 8A top) which also evoked the largest percentage of time deliberately exploring inside the vRoI. The video of the “princess” evoked a small shift in predictability during the first 04-05-12 session but in the following visit, during the 04-19-12 session the gain in predictability was higher and so was the % of time spent inside the vRoI. This reveals a gain that was not only retained but also enhanced 2 weeks later in the absence of additional practice sessions.

Consistent results are reported for a non-verbal boy with ASD in Figure 9. He, too, came to the realization on his own, without any verbal instructions, that (1) transient changes in media state were triggered by his hand; (2) by sustaining the hand inside the vRoI he could continuously watch the video of his preference. In his case, real-time self-videos were preferred according to the maximal step size in the Gamma plane of the stochastic signatures of the hand micro-rotations (from the brown asterisk representing the outside vRoI value to the brown dot representing the inside vRoI value). Likewise this is the media type that evokes the largest frequency of times that the hand visited inside the vRoI. Notice as well that as with the other participants in later sessions (e.g., from 03-23-12 to 04-13-12) there is a gain in predictability above and beyond its retention over time.

### Transient and long-term effects registered in all children

Besides transient positive effects during a single session, the experimental results indicated in each child a long-lasting longitudinal effect after 2 weeks with marked improvements from the earlier session (despite no practice during the period between sessions). In Figure 8 for example, the gains in predictability evoked by the “princess” video were retained from the 04-05-12 session to the 04-19-12 session. There was also a gain in predictability during the later 04-19-12 session as quantified by the shifts in the estimated parameters of the Gamma statistical distribution. On this later 04-19-12 session the “princess” video had a larger gain toward the Gaussian range of the Gamma plane (Figure 8B top) and the girl rotated her hand inside the vRoI for a larger percent of time than in the previous 04-05-12 session. This video without a doubt had a consistent positive longitudinal effect on the statistical signatures of the angular velocity-dependent variability of this participant.

In the example from the non-verbal boy with ASD, the real time self-video projected from the webcam facing him whenever the hand entered the vRoI had the largest shift (Figure 9B top) and led to the continuous and deliberate holding of the hand inside the vRoI. The “bluesclues” and “elmo” videos also shifted the stochastic signatures between OUT and IN the vRoI. The hand was exploring mostly outside the vRoI for the “bluesclues” and “elmo” videos (shown in Figure 9B bottom). This indicates that the search for the “magic spot” was not entirely random as indicated by the shifts in the stochastic signatures during this exploration outside the vRoI to more predictive ranges of the Gamma plane (Figure 9B top). As in the other children the second visit from 03-23-12 to 04-13-12 showed retention and improvement of the patterns, despite no practice sessions in the time between visits.

### Automatically assessing the quality of the session of the experimental intervention

The search patterns in real time for a given session were also informative of the quality of the session in terms of the learning stage. Goal-contacting sessions were those in which the search was conducive of intentionality, meaning that the goal was found and sustained the media playing most of the time. In this case the child deliberately held the hand inside the vRoI for the most part of the session in order to maximize the reward of continuously interacting with the media. Sessions that were mostly exploratory without success at figuring out the goal location were termed Goal-seeking sessions. The stochastic patterns could then reveal the level of randomness of predictability (systematicity) of the session and indicate if these were random motions or goal-seeking exploratory behaviors.

**Figure 10:**
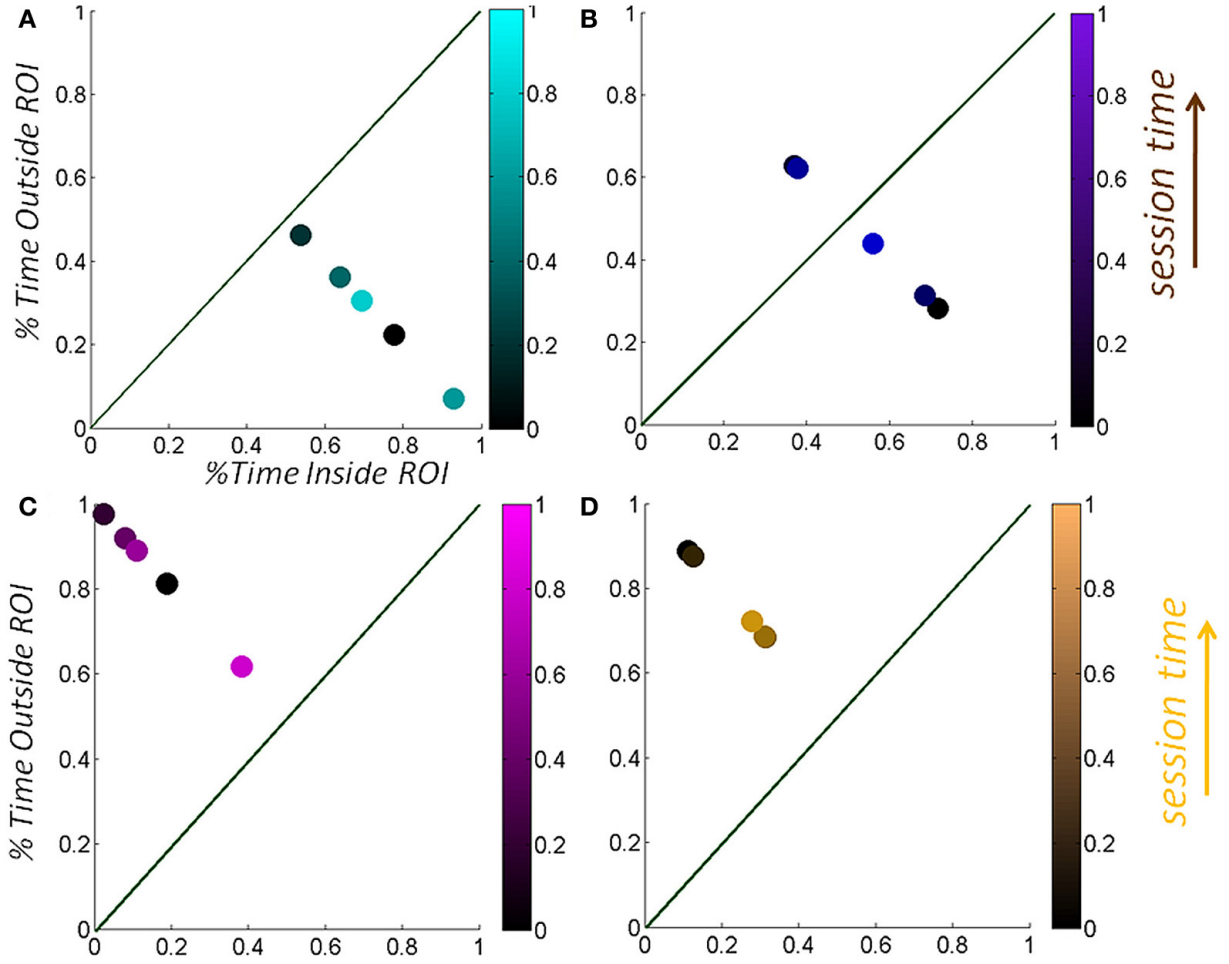
**Temporal progression of the validation metric as a function of session time-length. (A,B)** Quantification of the percentage of session time that the hand spent inside the vRoI (goal-contacting) vs. outside the vRoI (goal-seeking) **(C,D)** as a function of session length. Notice the progression of the bright colors toward the lower right as the recording goes on in **(C)** and **(D)**.

Examples of Goal-contacting sessions are shown on the top panels of the Figure 10. On the left panel the hand was maintained inside the vRoI the entire time while the right panel shows a transition from OUT to IN, as well as the intentional holding of the hand inside the vRoI as the session progresses in time (from darker to lighter colors). Examples of Goal-seeking sessions are shown on the bottom panels of Figure 10 where the hand was mostly exploring outside the vRoI. At the start of this session (darker dots) the patterns are most of the time far from the vRoI but as time progresses within the session (lighter colors) the hand is closer to the line of unity where the child visits with equal frequency the areas of interest IN *vs*. OUT of the vRoI.

### Adaptive capabilities are present in both TD and ASD children

The most interesting result of this work is that all participants with ASD showed proficiency in this task. They were able to detect the change that their motions caused in the state of the media. This change-detection capability was sufficient to spontaneously, without instructions, trigger a search in peripersonal space that moved from random to systematic and eventually intentional, as when the hand was deliberately held inside the vRoI in order to continuously play the media.

**Figure 11:**
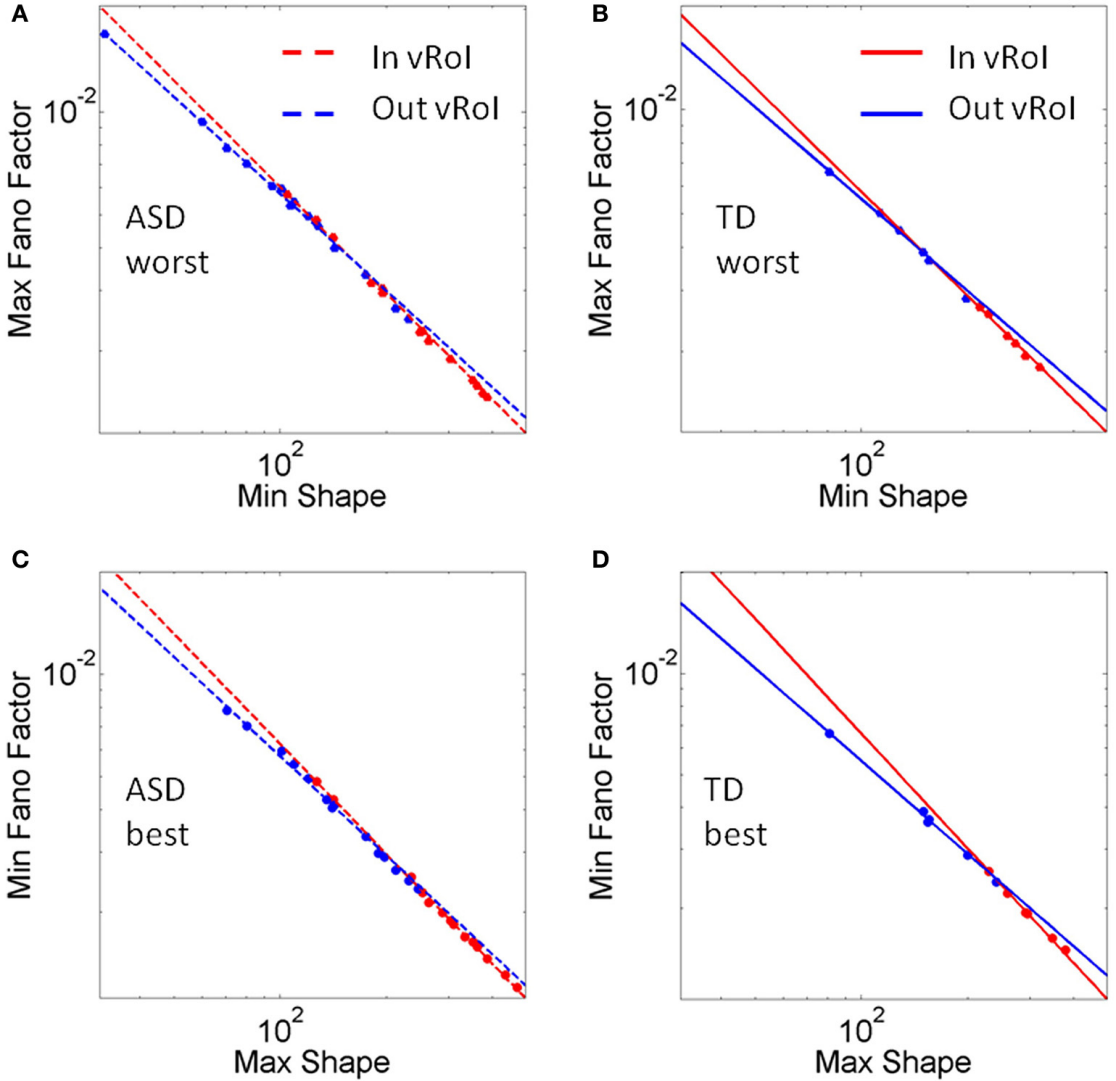
**Lack of differentiation between randomly searching motions and goal-directed motions in ASD. (A,B)** Worst performance of ASD and TD participants given by their highest Fano Factor values; lowest shape parameter values and poorest distinction between IN and OUT vRoI patterns (i.e., poor separation of the slopes). **(C,D)** Best performance in ASD and TD participants given by lower values of the Fano Factor, higher values of the shape parameter and larger separation of the slopes of the power relation (Table A4 lists the values of the power fit.)

This progression was captured in the evolution of their stochastic signatures of movement variability according to the statistical patterns of the angular rotations of the hand. This form of movement-based proprioceptive sensory input reshaped their behaviors and sustained their interest in the task. The Figure 6 shows that both TD and ASD participants improved their motion patterns by making them more reliable and predictable (shifted them down and to the right of the Gamma plane). Both groups came to discover on their own the goal(s) of the task and developed predictable and reliable statistics in their hand micro-movements. Their spontaneously emerging self-control resulted in motions with lower dispersion of the estimated probability distributions (Figures 6B,D) according to the Fano Factor which decreased in both the TD and the ASD participants when comparing the inside to the outside vRoI states and also when examining the gains over time (Figure 11 and Table A3).

An important result here which confirmed our previous findings in a larger group of ASD participants and under different experimental conditions (using open loop reaches; Torres et al., 2013), was that the subjects with ASD showed less discrimination between the patterns of spontaneous variations and those from intended motions. Figure 11 shows for a subset of the participants (each represented by a point) the changes in their performance across different sessions (we focus only on the participants that returned a month later) to compare their performance in terms of reliability and predictability of the estimated probability distributions.

Across all participants we obtained the worst (most unreliable and most random) and the best (most reliable and most predictable) performances. Figures 11A,B show the ASD and TD participants. These are the most random patterns (minimum values of the shape parameter) and the most unreliable distributions (the highest Fano factor denoting the largest dispersion given by the variance to the mean ratio). Notice that in both cases the distinction between the stochastic signatures of the random motions exploring outside the vRoI and those from the ones homing into the vRoI is larger in TD than ASD, but in both cases the slopes do tilt. In the best performance cases (Figures 11C,D) the slopes of the IN and OUT vRoI scatters begin to separate. This separation is more evident in TD participants. The difference in slope tilt shows a faster rate of change in the overall progression of the group towards the discrimination between variability patterns that come from spontaneous random and goal-seeking movements OUT vRoI, as well as patterns from the intentional goal-contacting movements IN vRoI. The former include random motions whereas the latter include very deliberate motions (as in the example of the child in Figure 9 lower panel, actually holding the hand IN vRoI to continuously play the media).

### SBV serves as a proxy to induce and sharpen IBV

Across all subjects the changes in the patterns of SBV associated with random motions in peripersonal space searching outside the vRoI supported the gains in predictability outside the vRoI. They were associated as well with changes in the patterns of intended behavioral variability (IBV) that the children acquired as they repeatedly aimed to the IN vRoI; and as they eventually made repeated contact with the goal, until they continuously sustained the hand at the goal. The motor variability associated with the spontaneous search movements (both random and goal-seeking) also accelerated implicit learning of secondary goals and their shifts in goal priority. These included: (1) shifting from crossing the vRoI in order to trigger the media ON; (2) shifting to actually holding the hand inside the vRoI so as to play the media continuously. As the patterns of SBV became more predictive so did the patterns of IBV. The latter variability was more directly associated with the achievement of the self-discovered goals. Table A4 lists for all subjects the Fano Factor and the change in predictability for both the SBV and the IBV linked respectively to the initially random and the acquired goal-directed motions.

## Conclusions and future steps

This work introduces a new concept that combines the notion of co-adapting in closed feedback loops the statistics of the real-time hand motions of the child and the statistics of the states of external stimuli. From these settings, with minimal to no instructions the child self-discovered the implicit goals of a task and naturally learned how to prioritize them in order to maximize the reward of the task. We were able to quantify in real time, with precise statistical indexes, the levels of predictability and reliability of their movements as they transitioned from random, to exploratory, to goal-seeking, and goal-contacting motions until they turned into intended anticipatory behavior.

We quantified the transitions from goal-directness to intentionality when the children deliberately held their hand in the vRoI in order to continuously play the media. This progression towards connecting the child's actions and intentions occurred in a matter of seconds within one session. The transient shifts in stochastic patterns of one session were retained weeks later and even improved without practicing the task during the time period between visits. The children independently drove the flow of the experimental intervention as the real-time feedback from their movements helped them self-discover cause and effect between the statistics of their hand motions and those of the media states. The child's self-discovery of such relations unlocked the volitional control of his/her actions and helped them modulate the position and orientation of the hand in space so as to sustain the media playing to reliably maximize this rewarding outcome.

This closed loop concept is rooted in the basic Brain Machine (Computer) Interface paradigms, (BMI or BCI; Vidal, 1973). The novelty in our approach is that instead of tracking/adapting a central neural signal to control an external device; the present experimental intervention co-adapts the real time statistical signals recorded from the peripheral physical micro-movements of the body with those statistical patterns reflecting the state of the external media. The methods use both statistics (externally and internally driven) as feedback signals to modify the stochastic patterns of the child's behavior. In this regard the statistical patterns from the periphery (the limbs movements) are continuously feeding back into the central centers of the brain via afferent channels. This continuous flow of re-afferent peripheral information harnessed from the motor behavior was systematically used here as a proxy to evoke better modulation of the central control of their motor patterns. The child learned to better regulate and eventually anticipate centrally sent efferent motor signals according to the patterns of peripheral hand movement variability, which we could read out in real time. These patterns transformed from random and noisy to predictable and reliable in a matter of minutes.

Under these settings the non-verbal children with ASD became engaged in the task, improved the autonomy over their limb-hand linkage and self-discovered the implicit goals and the hidden priorities that the experimenter defined. All children resolved the very problem that they self-discovered. In tandem they shifted the stochastic signatures of their hand micro-motions from random, noisy, and restricted to predictive and reliable with higher explorative bandwidth. Moreover, these positive gains were retained over time despite no training during the intermediate weeks.

We used a new SPBA (Torres and Jose, 2012). This new platform permits the real-time objective dynamic tracking of the non-stationary statistical features of the continuous flow of natural behaviors. We can thus during a session, detect shifts in their stochastic trajectories as a function of preferred forms of sensory guidance, context, etc. The term “preferred” in this case is revealed by the maximal shift toward the formation of a motor expectation (predictive and reliable). That maximal shift tells the forms of sensory guidance, context, etc. causing maximal rates of change in the stochastic toward the desirable statistical regimes.

The combination of this new experimental paradigm concept (encouraging spontaneous self-discovery of the goals) with the SPBA enables the automatic assessment of the continuous flow of natural behaviors. It also enables the discovery and real-time tracking of exploratory patterns in the children. These patterns in the present settings evolved from random motions to deliberate trial-and-error, then to goal-directed motions and finally to intentional actions. Importantly we were able to automatically select the media type that most likely accelerated this learning process, based on this real-time automatic tracking.

All the children with ASD had goal-directed behaviors, a fact that is currently used by behavioral approaches reinforcing such behaviors through commands and explicit goal-directed instructions. Such therapeutic regimes have provided a working platform for early interventions and treatments. In some cases the child visibly changes and can be mainstreamed into public or private schools hosting TD peers. Without a doubt behavioral therapies are very important. Whether relaxed (music therapy, horse therapy, dancing therapy, etc.) or structured (speech therapy, occupational therapy, physical therapy, Applied Behavioral Analyses, rapid prompting methods, etc.), these therapies, each one in its own right, have played and are bound to continue playing a critical role in the treatments of some autism type and in general research. Nonetheless, two aspects have been lacking in all methods: objective quantification and assessment of spontaneous aspects of the behavior, occurring largely beneath awareness. Furthermore, their reliance on explicit instructions when the individual with ASD—particularly the non-verbal individual—may not be able to follow instructions on command; their reliance on stimuli that is inferred by the clinician to be the best for the child without methods for blind validation; their general reliance on observation and hand-written scores and their overall subjective tracking methods call for a dramatic change in traditional therapeutic regimes. With the advent of current computational technological advances and algorithms these therapies can do better. They already have in place the infrastructure necessary to provide the means to revolutionize the ways in which ASD is treated and tracked over time. But they need major changes for a truly optimal and effectively reproducible outcome that could potentially uncover universal principles invariably leading to success in ASD interventions. The need for objective, automatic, computerized methods has been imminent for quite some time now. Such methods would provide “the neutral outsider” to anyone's agenda and biases, and would help reconcile senseless controversies in autism exclusively based on opinion and observations.

Our results show improvements with retention over time. They call for a major transformation in the philosophy of the current therapeutic interventions in ASD and in the rigor and objectivity with which the outcome of such methods are currently assessed. The methods presented in this report provide a precise prescription to achieve positive changes toward anticipatory behaviors. The results also invite the field to shift from being exclusively command/instruction driven to allow the person with ASD to spontaneously explore and self-discover the purpose(s) of a given task whenever possible.

Every individual in our study, independent of the degree of verbal capabilities and reported IQ score, was capable of performing this implicitly defined task with minimal to no instruction. By engaging their sensory motor systems and implicitly driving the child with the external input we were able to close the corrupted feedback loops and sensory-substitute the noisy-random-restricted peripheral limb's motions (which are a form of continuous kinesthetic feedback) with the external media of their liking. In this augmented physical reality setting, as the children embodied the statistics of the external media states, they self-discovered cause and effect relations. This self-realization prompted predictive statistical regimes in the media states that helped reinforce the volitional control over their own motor actions in closed loop with the media.

The children with ASD in this study developed more reliable, anticipatory motor statistics that were retained and even improved weeks later even without practice. More importantly we could backtrack exactly which media type was the most effective in the acquisition of precise indexes of reliability and predictability and reconstruct for each child the path of least resistance during this experimental intervention: the path with the fastest rate-of-change toward anticipatory behavior. We could do so for both intentional segments of their behavior and for co-existing spontaneous segments as well. The results reinforce the notion that beneath our awareness other aspects of our behaviors are co-occurring with the deliberate aspects and revealing fundamental information about the learning process. Under the presently proposed concept we can automatically and objectively register and dynamically track such implicit changes in parallel with the changes in deliberate control (Torres, 2011, 2012, 2013; Torres et al., 2013).

We have discovered here a way to (1) engage individuals with autism in spontaneous exploration; (2) modulate the peripheral motor output as a function of external stimuli statistics; (3) extract the form of sensory guidance that most likely accelerates shifts toward predictive and reliable statistical regimes of motor behavior; (4) make the gains long-term rather than transient; (5) automatically backtrack the learning trajectories of deliberate and spontaneous aspects of the behavior as well as their precise rates of change—unique to each child. All of it could be done in real time and checked again longitudinally in a novel way where the child, rather than the therapist/researcher, was the leading party. All throughout the session, the researcher merely intervened to gently steer the child's self-discovery process. This is in stark contrast with the therapist/researcher assuming the leading role. The latter is routinely done in current approaches to ASD treatments and research. In this regard, the variability from the spontaneous segments of the behavior played a fundamental role in the self-discovery process that the children underwent.

The spontaneous motor variability patterns from the periphery, which are currently not tracked in traditional behavioral interventions, turned out to be critical in order to evoke and sustain centrally driven intentionality and autonomous control in the actions of these non-verbal children with ASD. Here we pose the question to the field of whether the sensory feedback from peripherally driven changes could systematically impact the development of central centers of the brain. We further ask whether in ASD the levels of gain-retention would be better and more effective in some children when the interventions were based on spontaneous self-discovery rather than exclusively based on explicit commands.

We propose that sub-cortically based and peripherally based anticipatory control of movement may typically develop along different time scales than anticipatory control from the neo-cortex. It is known that philogenetically there is an order of appearance in the development of such structures that evolution has imposed (Porges, 2003). Furthermore, recent neuromagnetic developmental studies on motor anticipation during button presses have revealed that between the 4 and 6 years of age TD children do not yet have the patterns of anticipatory motor control that they later on develop by 12 years of age (Gaetz et al., 2010). In contrast we have recently found that in TD children the statistical signatures of anticipatory motor control patterns from the peripheral limbs are already in place after 4 years of age (Torres et al., 2013). Future research to address these issues during typical development is warranted in our lab: (1) whether there is an order of appearance for statistically anticipatory motor control, for example, starting at the peripheral synapses, following at the sub-cortical structures, and later appearing in the neocortex; (2) whether peripheral feedback can be used to reshape central structures during development; (3) whether spontaneous self-discovery evokes, sustains, and modulates intentional control. Finally we plan to investigate how anticipatory motor control evolves in ASD upon an early developmental glitch that may fundamentally alter the order of systemic maturation and result in a very different form of adaptive cognition, one which we cannot at present access or even know how to begin defining.

This new conceptual paradigm and statistical platform are simple and easy to use. They go well with current computational technological advancements and complement in non-trivial ways the present approaches to autism research and treatments. These methods are inclusive of the self-discovery abilities and sensory strengths of the individual with ASD. We invite others to try them out and unleash the potential of all the children according to the sensory-motor capabilities and predispositions that they already have. As Esther Thelen taught us in her seminal work (Thelen and Smith, 1994), p. 305, “*Development does not happen because internal maturation processes tell the system how to develop. Rather, development happens through and because of the activity of the system itself*.”

### Conflict of interest statement

The authors declare that the research was conducted in the absence of any commercial or financial relationships that could be construed as a potential conflict of interest.

## Figures and Tables

**Table A3:**
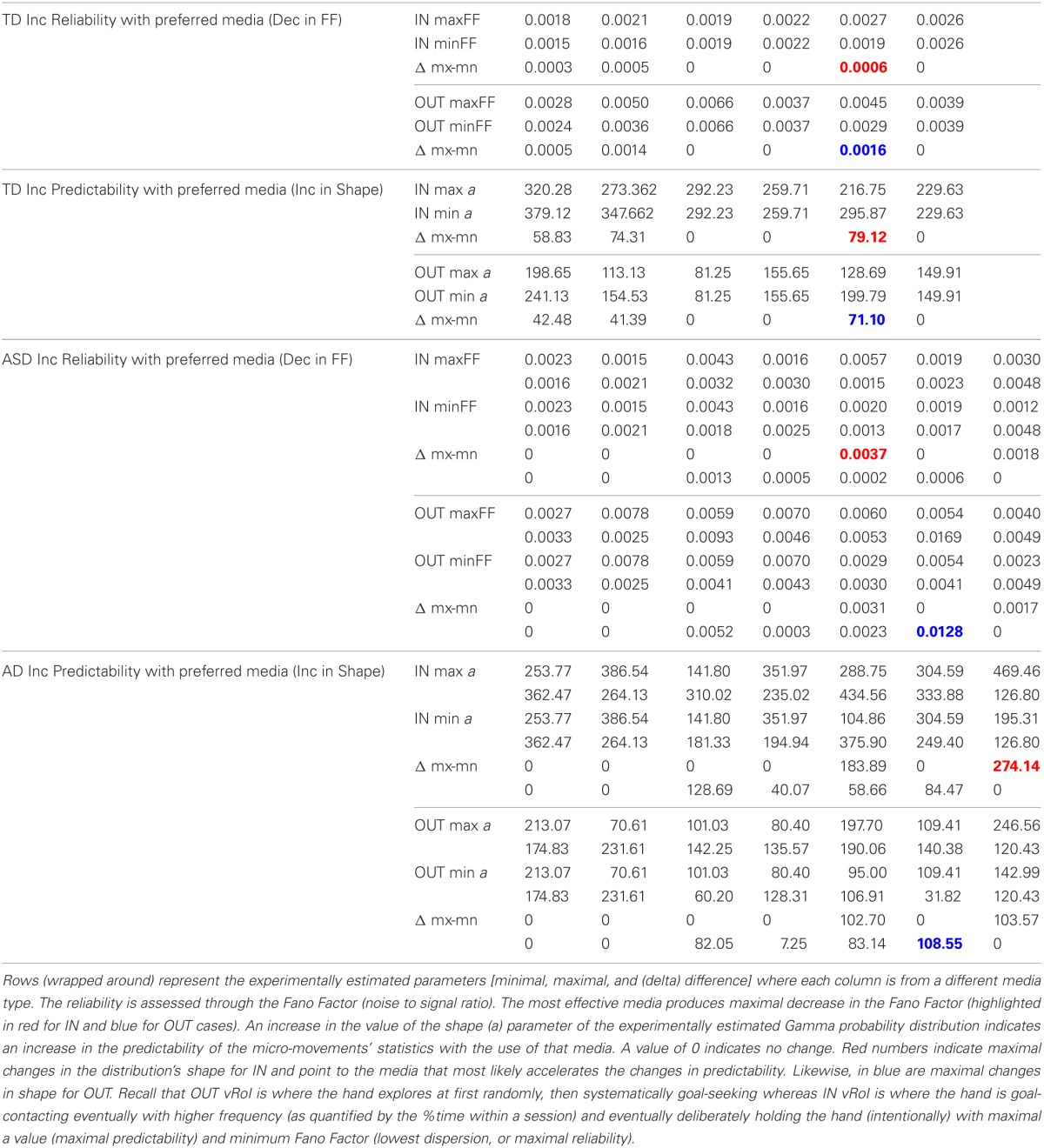
**Reliability and Predictability of the experimentally estimated probability distributions for TD and ASD cases to assess the effectiveness of different media types**.

Rows (wrapped around) represent the experimentally estimated parameters [minimal, maximal, and (delta) difference] where each column is from a different media type. The reliability is assessed through the Fano Factor (noise to signal ratio). The most effective media produces maximal decrease in the Fano Factor (highlighted in red for IN and blue for OUT cases). An increase in the value of the shape (a) parameter of the experimentally estimated Gamma probability distribution indicates an increase in the predictability of the micro-movements' statistics with the use of that media. A value of 0 indicates no change. Red numbers indicate maximal changes in the distribution's shape for IN and point to the media that most likely accelerates the changes in predictability. Likewise, in blue are maximal changes in shape for OUT. Recall that OUT vRoI is where the hand explores at first randomly, then systematically goal-seeking whereas IN vRoI is where the hand is goal-contacting eventually with higher frequency (as quantified by the %time within a session) and eventually deliberately holding the hand (intentionally) with maximal a value (maximal predictability) and minimum Fano Factor (lowest dispersion, or maximal reliability).

## Appendix

**Table A1:** **Scores of the participants with ASD reported from independent clinical assessments by licensed clinicians**.

			**Stanford–Binet**			**ADOS scores/GARS scores**							
**No.**	**Gender**	**Age (yrs)**	**NVIQ**	**VIQ**	**FSIQ**	**Stereo**	**Com**	**Soc**	**Com + Soc**	**Stereo SS**	**Com SS**	**Soc SS**	**Autism index**
1	F	5.9	44	51	45	2	4	13	17	N/A	N/A	N/A	N/A
2	M	13.8	42	43	40	N/A	N/A	N/A	N/A	N/A	N/A	N/A	N/A
3	M	7.6	50	46	45	3	6	11	17	N/A	N/A	N/A	N/A
4	F	7.8	42	43	40	4	7	12	19	N/A	N/A	N/A	N/A
5	M	9.9	42	43	40	4	5	8	13	N/A	N/A	N/A	N/A
6	M	10	N/A	N/A	107	3	3	9	12	N/A	N/A	N/A	N/A
7	M	10.3	42	43	40	3	4	10	14	N/A	N/A	N/A	N/A
8	M	11.5	100	82	90	7	5	6	11	N/A	N/A	N/A	N/A
9	F	11.5	50	43	44	N/A	N/A	N/A	N/A	N/A	N/A	N/A	N/A
10	M	11.7	42	43	40	5	8	10	18	N/A	N/A	N/A	N/A
11	M	11.7	43	43	40	N/A	N/A	N/A	N/A	N/A	N/A	N/A	N/A
12	M	12	N/A	N/A	67	4	5	13	18	N/A	N/A	N/A	N/A
13	F	12	N/A	N/A	60	4	8	10	18	N/A	N/A	N/A	N/A
14	M	12	N/A	N/A	95	2	5	8	13	N/A	N/A	N/A	N/A
15	M	13	N/A	N/A	89	2	3	7	10	N/A	N/A	N/A	N/A
16	M	14	N/A	N/A	74	3	9	10	19	N/A	N/A	N/A	N/A
17	F	14.3	50	43	44	N/A	N/A	N/A	N/A	8	11	9	124
18	F	15	N/A	N/A	71	6	5	7	12	N/A	N/A	N/A	N/A
19	F	15.8	42	43	40	N/A	N/A	N/A	N/A	13	10	11	109
20	F	16	N/A	N/A	81	2	7	9	16	N/A	N/A	N/A	N/A
21	M	18	N/A	N/A	101	2	4	6	10	N/A	N/A	N/A	N/A
22	M	18	N/A	N/A	96	4	4	8	12	N/A	N/A	N/A	N/A
23	M	25	N/A	N/A	99	6	3	7	10	N/A	N/A	N/A	N/A
24	M	13.8	42	43	40	N/A	N/A	N/A	N/A	N/A	N/A	N/A	N/A
25	M	9.0	42	44	40	1	5	10	15	N/A	N/A	N/A	N/A

**Table A2:** **TD participants**.

**Number**	**Gender**	**Age (Yrs)**
1	M	3.3
2	M	3.3
3	F	3.8
4	M	3.8
5	F	4.0
6	M	4.0
7	M	4.8
8	M	5.0

**Table A4:** **Goodness of fit parameters of the power fits in Figure 11 for the worst (lowest reliability and predictability) and best (highest reliability and predictability) of the micro-movements' statistics as they change in tandem with the media states statistics IN and out of the vRoI for both the TD and ASD children**.

	**TD**	**ASD**
Max FFI vs. Min ShI	General model Power1:	General model Power1:
	*f (x) = a * x∧b*	*f (x) = a * x∧b*
	Coefficients (with 95% confidence bounds):	Coefficients (with 95% confidence bounds):
	*a* = 0.5755 (0.1678, 0.9833)	*a* = 0.7014 (0.5873, 0.8155)
	*b* = −0.9987 (−1.127, −0.8707)	*b* = −1 .033 (−1.065, −1.001)
	Goodness of fit:	Goodness of fit:
	SSE: 4.459e-009	SSE: 5.114e-008
	*R*-square: 0.9926	*R*-square: 0.9978
	Adjusted *R*-square: 0.9907	Adjusted *R*-square: 0.9976
	RMSE: 3.339e-005	RMSE: 6.528e-005
Max FFO vs. Min ShO	General model Power1:	General model Power1:
	*f (x) = a * x∧ b*	*f (x) = a * x∧b*
	Coefficients (with 95% confidence bounds):	Coefficients (with 95% confidence bounds):
	*a* = 0.3201 (0.1911, 0.449)	*a* = 0.4389 (0.405, 0.4728)
	*b* = −0.8819 (−0.9667, −0.7971)	*b* = −0.9414 (−0.9601, −0.9226)
	Goodness of fit:	Goodness of fit:
	SSE: 3.945e-008	SSE: 2.055e-007
	*R*-square: 0.9954	*R*-square: 0.9988
	Adjusted *R*-square: 0.9942	Adjusted *R*-square: 0.9987
	RMSE: 9.931e-005	RMSE: 0.0001309
Min FFI vs. Max ShI	General model Power1:	General model Power1:
	*f (x) = a * x∧ b*	*f (x) = a * x∧b*
	Coefficients (with 95% confidence bounds):	Coefficients (with 95% confidence bounds):
	*a* = 1.226 (0.5232, 1.929)	*a* = 0.8705 (0.8061, 0.9349)
	*b* = −1.134 (−1.236, −1.033)	*b* = −1 .073 (−1.087, −1.059)
	Goodness of fit:	Goodness of fit:
	SSE: 3.344e-009	SSE: 7.414e-009
	*R*-square: 0.9956	*R*-square: 0.9995
	Adjusted *R*-square: 0.9945	Adjusted *R*-square: 0.9995
	RMSE: 2.891e-005	RMSE: 2.486e-005
Min FFO vs. Max ShO	General model Power1:	General model Power1:
	*f (x) = a * x∧ b*	*f (x) = a * x∧b*
	Coefficients (with 95% confidence bounds):	Coefficients (with 95% confidence bounds):
	*a* = 0.3734(0.2854, 0.4613)	*a* = 0.4918 (0.384, 0.5995)
	*b* = −0.9169 (−0.9658, −0.868)	*b* = −0.9666 (−1.013, −0.92)
	Goodness of fit:	Goodness of fit:
	SSE: 1.765e-008	SSE: 2.184e-007
	*R*-square: 0.9984	*R*-square: 0.9944
	Adjusted *R*-square: 0.998	Adjusted *R*-square: 0.9939
	RMSE: 6.642e-005	RMSE: 0.0001349
